# Hypoxia: syndicating triple negative breast cancer against various therapeutic regimens

**DOI:** 10.3389/fonc.2023.1199105

**Published:** 2023-07-10

**Authors:** Nityanand Srivastava, Salman Sadullah Usmani, Rajasekaran Subbarayan, Rashmi Saini, Pranav Kumar Pandey

**Affiliations:** ^1^ Department of Cell Biology, Albert Einstein College of Medicine, Bronx, NY, United States; ^2^ Department of Molecular Pharmacology, Albert Einstein College of Medicine, Bronx, NY, United States; ^3^ Department of Radiation Oncology, Albert Einstein College of Medicine, Bronx, NY, United States; ^4^ Research, Chettinad Hospital and Research Institute, Chettinad Academy of Research and Educations, Chennai, India; ^5^ Department of Zoology, Gargi College, University of Delhi, New Delhi, India; ^6^ Dr. R.P. Centre for Opthalmic Sciences, All India Institute of Medical Sciences, New Delhi, India

**Keywords:** Hypoxia, HIF-1, TNBC, immune escape, DNA damage response, chemotherapy, immunotherapy, cancer vaccines

## Abstract

Triple-negative breast cancer (TNBC) is one of the deadliest subtypes of breast cancer (BC) for its high aggressiveness, heterogeneity, and hypoxic nature. Based on biological and clinical observations the TNBC related mortality is very high worldwide. Emerging studies have clearly demonstrated that hypoxia regulates the critical metabolic, developmental, and survival pathways in TNBC, which include glycolysis and angiogenesis. Alterations to these pathways accelerate the cancer stem cells (CSCs) enrichment and immune escape, which further lead to tumor invasion, migration, and metastasis. Beside this, hypoxia also manipulates the epigenetic plasticity and DNA damage response (DDR) to syndicate TNBC survival and its progression. Hypoxia fundamentally creates the low oxygen condition responsible for the alteration in Hypoxia-Inducible Factor-1alpha (HIF-1α) signaling within the tumor microenvironment, allowing tumors to survive and making them resistant to various therapies. Therefore, there is an urgent need for society to establish target-based therapies that overcome the resistance and limitations of the current treatment plan for TNBC. In this review article, we have thoroughly discussed the plausible significance of HIF-1α as a target in various therapeutic regimens such as chemotherapy, radiotherapy, immunotherapy, anti-angiogenic therapy, adjuvant therapy photodynamic therapy, adoptive cell therapy, combination therapies, antibody drug conjugates and cancer vaccines. Further, we also reviewed here the intrinsic mechanism and existing issues in targeting HIF-1α while improvising the current therapeutic strategies. This review highlights and discusses the future perspectives and the major alternatives to overcome TNBC resistance by targeting hypoxia-induced signaling.

## Graphical Abstract

Hypoxia within the TNBC tumor microenvironment stabilizes the HIF-1α to decide the cancer cell fate, genetics, metabolism, immune response and clinicopathology. (Created via BioRender.com).

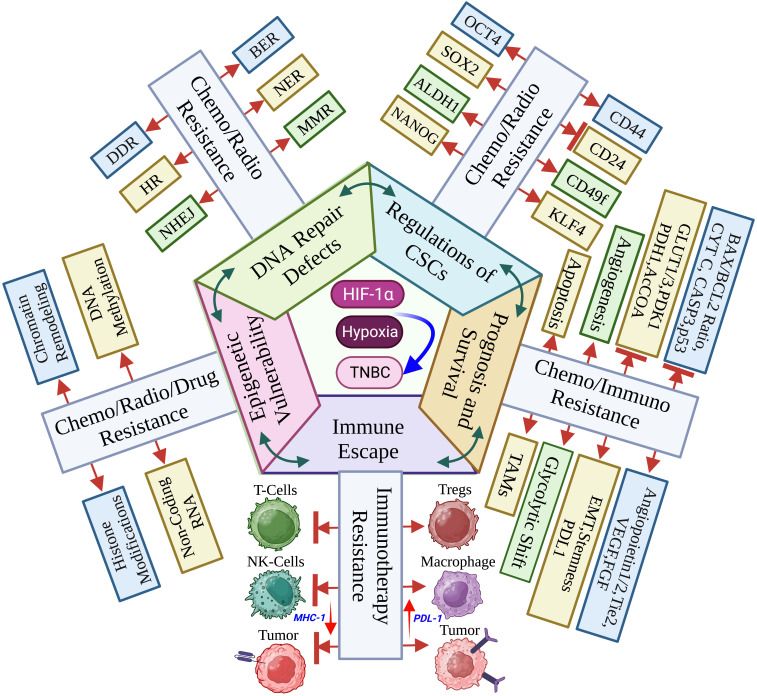

## 1 Introduction

Breast cancer (BC) is the 2^nd^ most common leading cause of cancer-related deaths, mostly diagnosed in young women. It accounts for over 43,000 estimated deaths annually among women in the US alone (1, 2). The stratification of breast carcinoma involves histological features, including the expression of markers such as estrogen receptor (ER) (3), progesterone receptor (PR) (4), and human epidermal growth factor receptor 2 (hEGFR2) (5). Furthermore, six intrinsic subtypes of TNBC, such as basal like, HER2 enriched, luminal A, luminal B, normal like and claudin low have been identified by high-throughput transcriptomic and genomic sequencing (6). These subtypes display distinct features in molecular portraits as well as clinical outcomes (5, 7, 8). TNBC is the small claudin-low subset of BC. It has a high histological grade, high epithelial-mesenchymal transition (EMT) marker enrichment, and high metastasis rates, including aggressive cancer stem cell-like features. In addition, they also have low luminal differentiation power and low expression of cell-cell adhesion molecules but are highly hypoxic in nature, making TNBC the most aggressive and deadliest subtype of BC (9–12). The term “negative” in TNBC refers to a very uncommon BC subtype that does not express ER, PR, and hEGFR2 (11, 13–16).

Epidemiological data analysis reveals that premenopausal women under the age of 40 are the primary suspects of TNBC occurrence, and approximately 20% of all BC patients are under the young age (13, 14). The TNBC patient’s survival time is comparably shorter than that of patients with another subtype of BC. The mortality rate of TNBC patients is also significantly high, and around 40% of deaths occur in TNBC patients within five years after the first diagnosis (13, 15). TNBC is a highly heterogenous subtype, and because of its aggressiveness and invasiveness, approximately 46% of TNBC patients have distant metastasis. Patients diagnosed with TNBC are more likely to develop distant metastasis within three years of diagnosis. Besides, the overall survival rate of metastatic patients is low, and based on the available data, the average median survival is only 13.3 months. The studies also demonstrate that the chances of tumor recurrence after surgery are as high as 25%. Brain and visceral organ metastasis also have been reported in metastatic TNBC patients. Most distant metastasis happen in the third year following diagnosis (16–18). TNBC patients have a shorter average time to relapse (19–40 months) than non-TNBC patients (35–67 months). According to published statistics, the death rate of TNBC patients after tumor recurrence is as high as 75% compared to non-TNBCs (16, 19).

Since heterogeneity, aggressiveness, and hypoxia create a favourable microenvironment for TNBC to grow and spread faster than other types of invasive BC, therefore, planning an effective treatment strategy for TNBC patients’ remains a herculean task. Although, in recent years, much research has been focused on identifying the specific targets of TNBC, the need for well-defined molecular targets in TNBC has resulted in limited therapeutic options. Currently, TNBC patients treatment mainly relies on standard therapies for TNBC, such as surgery, chemotherapy (CT), radiotherapy (RT), and photodynamic therapy (PDT) (20, 21). Therefore, identifying new therapeutic targets for TNBC is an urgent need and a high priority for society. Studies have investigated and identified several therapeutic molecules targeting oncogenic signaling pathways, including the PI3K/AKT/mTOR pathway and Src/Wnt signaling, to check their effectiveness in treating TNBC (22, 23). In addition, the alterations of BC genes 1 and 2 (BRCA1/2) and DNA damage-responsive (DDR) genes, including dysfunction of epigenetic and immune regulators, have also been used as an inhibitory index to predict treatment response in TNBCs (13, 24). Moreover, several studies also demonstrated a promising result by following a combination drug therapy strategy where they use targeted cancer drugs combined with chemotherapy or radiotherapy, and a few are in clinical trials (25, 26). Although the current treatment strategies are significantly effective, unfortunately, the overall treatment outcomes are highly variable, and it could be because of the highly heterogeneous nature of TNBC. Therefore, there is an urgent demand for alternative and accurate therapeutic strategies with improved efficiency, either alone or in combination with other therapies. HIF-1 is a key heterodimeric transcription factor of hypoxia. It consists of an oxygen-sensitive α subunit and a constitutively expressed β subunit. It is the master regulator to induce oncogenes and inactivate tumor suppressor genes functionality. It is widely regulated by inflammatory mediators released by tumor stromal cells TNBC that allow cellular adaptation against hypoxia (27, 28). Several studies established and proved that an intra-tumoral hypoxic environment creates a negative impact on the survival of BC patients and is associated with tumor aggressiveness and heterogenic phenotypes, which further induce a high risk of metastasis and provide a shielding barrier against various therapies such as chemotherapy, radiotherapy, and immunotherapy which suggest that hypoxia makes TNBC resistant to different treatments (28–31). Available evidence also supports the hypothesis that the elevation of HIF-1α expression in TNBC may provide a suitable environment for TNBC to grow in hypoxic conditions (32). Therefore, targeting hypoxic cancer cells seems to be a plausible idea for treating TNBC. Studies have also revealed that in TNBC, HIF-1α regulates the various complex biological processes and activates the transcription of several target genes involved in regulating angiogenesis, cellular metabolism, stem cell differentiation, and immune cell migration. The activation of these pathways further induces the expression of downstream gene products associated with stemness and EMT that have been further proven to be hyperactivated in TNBC by various research groups (33–35). Therefore, targeting HIF-1α could be a significant potential therapeutic option.

The pattern of HIF-1 expression in TNBC as well as the mechanism by which HIF-1 accelerates the disease are reviewed. This review also examines how breast cancer stem cell (BCSC) enrichment and immune evasion are affected by HIF-1 in the regulation of angiogenesis, invasion, and metastasis. It has also been investigated how HIF-1 affects TNBC through chemotherapy, immunotherapy, anti-angiogenic therapy, adjuvant therapy, PDT, adoptive cell therapy, antibody drug conjugates, cancer vaccines and also in combination therapies. The internal mechanisms as well as prospective therapeutic medicines that target HIF-1 are also reviewed.

## 2 The linkage between TNBC and hypoxia

The hypoxia-related mechanism is one of the distinguishing features of the cancer signaling system (34, 36). Each stage of the metastatic process is constrained by the hypoxic tumor microenvironment, which also regulates different cancer phenotypes (31, 37). Intratumorally, hypoxia is the major critical microenvironmental factor that is associated with TNBC and its invasiveness, metastasis and mortality (38). Additionally, disorganization of the tumor vasculature during tumor growth, is also associated with fluctuation of oxygen and glucose levels, leading to a heterogenous state of hypoxia, aerobic and anaerobic glycolysis (39, 40). Therefore, it is fundamental to correctly define the term ‘hypoxia’, presumed as an unusual system accompanying advanced malignancy by absolutely different mechanisms like chronic permanent inflammation or cell death pathways. Yet, the local hypoxia is deliberately produced by tumor cells to induce angiogenesis, hence directing the growth factors (30, 41). In chronic hypoxia, cells remain in a state above the diffusion limit of oxygen due to increased distance caused by tumor expansion (42, 43). This oxygen fluctuation within the tumor stipulates cancer cells for both aerobic and anaerobic glycolysis (44). Amplified glycolysis with or without oxygen is an important indicator for cancer and serves as a connecting link between TNBC and hypoxia (45, 46). Hypoxic microenvironment response in TNBC is tightly regulated by HIFs, which contain either HIF-1α or HIF-2α with a constituent expression of the HIF-1β subunit. Their elevation is associated with an increased risk of metastasis and mortality (34, 47). HIF-1α, HIF-2α, or both cause the activation of hypoxia-inducible genes, and their translational product is involved in several steps of the TNBC invasion and metastasis (48, 49). Under normoxic conditions, HIF-1α subunits are finally degraded by the proteosome, whereas hypoxia inhibits prolyl hydroxylases (PHD) and factor-inhibiting HIF-1 (FIH-1), key components required in the steps involved in the proteasomal degradation of HIF-1α, leading to HIF-1α stabilization and translocation to the nucleus, where they dimerize with HIF-1β and finally bind with hypoxia response elements within the promoters of target genes (50–52). In TNBCs, HIF target genes are highly expressed, whereas the expression of progesterone, estrogen and human epidermal growth factor receptors are deficient. Thus, TNBCs respond poorly to several current therapeutic regimens (53, 54). Giatromanolaki et al. claimed that the overexpression of HIF-1α is closely related to the immune response and adverse prognosis of BC and also inhibits the proliferation and survival of cytotoxic T cells and the expression of IL-2 and IFN-γ cytokines (55).

### 2.1 TNBC hypoxia in relation with prognosis and survival

In TNBC, hypoxia plays a critical role in the prognosis and survival of cancer cells *via* the mediation of angiogenesis, glycolytic shift, apoptosis, and the recruitment of tumor-associated macrophages (TAMs). Hypoxic conditions upregulate the angiogenic growth factors and their receptors, leading to increased vascular permeability due to endothelial cell migration, and thus mediating TNBC angiogenesis. This phenomenon of angiogenesis is achieved *via* HIF pathways by regulating several pro-angiogenic genes such as angiopoietin-1 and 2, tunica intima endothelial kinase 2 (Tie2), vascular endothelial growth factor (VEGF) and their receptor, platelet-derived growth factor (PDGF), fibroblast growth factor (FGF), etc. (56). Besides, hypoxia causes the accumulation of TAMs, exhibiting the cancerous phenotype. TAMs also secrete angiogenic growth factors, leading to angiogenesis and prognosis (57, 58).

Hypoxia induces apoptosis by regulating several pro- and anti-apoptotic pathways either by HIF-dependent or independent mechanisms. Hypoxia reduces the bax/bcl-2 ratio as well as cytochrome c release and caspase-3 activity, thus inhibiting the pro-apoptosis pathways. In addition, hypoxic conditions favor the selection of p53 mutant cells having elevated levels of bcl-2, which is a well-known inhibitor of apoptosis, thus causing the decline in the p53 and bcl-2 ratio (p53/bcl-2), which further increases the mutation rates in clone populations (59, 60). This endless cycle promotes the prognosis of TNBCs (61).

**Figure 1:**
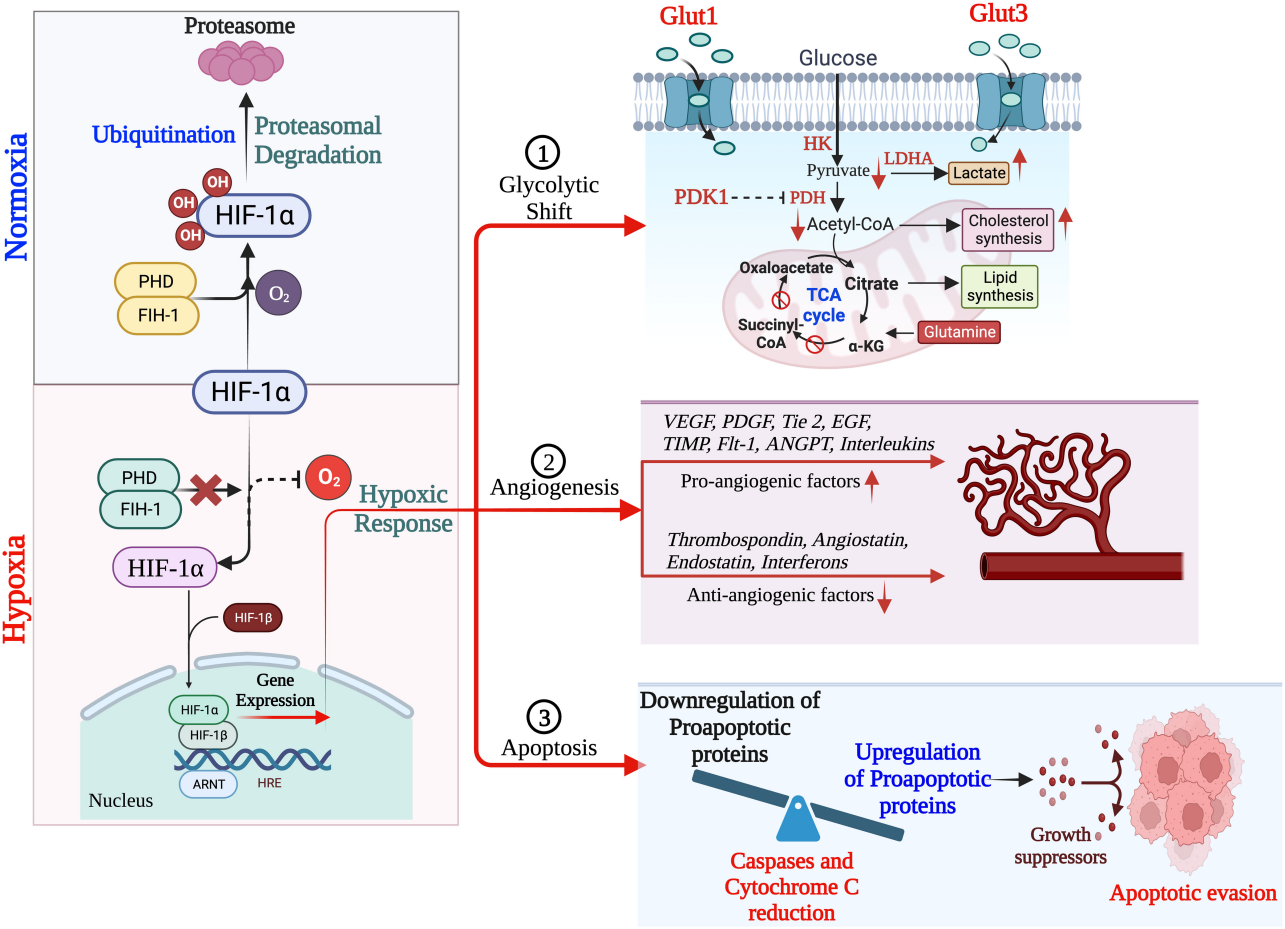
Schematic overview of HIF-1α regulation leading to hypoxia, resulting into adaptation of metabolic pathways, inducing angiogenesis and apoptosis. (Created via BioRender.com).

Hypoxia modulates the expression of glucose transporters like GLUT-1 and GLUT-3 as well as glycolytic enzymes such as hexokinase 1, hexokinase 2, and phosphoglycerate kinase 1 (PDK1), causing a glycolytic shift from oxidative phosphorylation to glycolysis. HIF-1α plays a fundamental role in this metabolic adaptation (62). HIF-1α induces the expression of PDK1, which after phosphorylation inhibits pyruvate dehydrogenase (PDH), a key enzyme converting pyruvate to acetyl-CoA. In anaerobic glycolysis, this pyruvate is forced to be metabolized into lactate. Thus, hypoxic conditions reduce the amount of acetyl-CoA available to enter the Krebs cycle or TCA cycle, leading to a reduced amount of substrate availability for mitochondrial respiration as well as oxygen consumption (63–65). It is a well-known fact that cancer cells immediately use glycolysis, even when sufficient oxygen is available. This dependency on inefficient aerobic glycolysis is known as the Warburg effect, which promotes tumor prognosis and survival (**Figure 1**).

### 2.2 Hypoxia in regulation of cancer stem cells in TNBC

CSCs are a small heterogenous subset with self-renewal characteristics. These cells have a tremendous power to differentiate into all other specific cell types within the tumor tissues and can survive after therapy (66, 67). Specific biomarkers such as CD44^high^/CD24^low^, CD49f, and aldehyde dehydrogenase 1 (ALDH1) define the BCSCs in TNBC and are predominantly associated with a poor survival rate in TNBC patients (68, 69). Several analytical reports on human breast carcinomas have proved that CD44^high^CD24^low^ALDH1^high^ CSCs predominantly associated with TNBCs and are significantly associated with tumor recurrence (69, 70). The hypoxia environment in TNBCs induces several stress-responsive genes which modulate the CSCs to activate their self-renewal and anti-apoptotic phenotype properties. These properties play a crucial role in tumor growth, immune evasion, metabolic reprogramming, drug resistance, and constraining clinical outcomes by modulating the transcription of several target genes (71). Hypoxia-activated pathways in the tumor microenvironment, such as HIF, CD133, CD24, CD47, DLK1, and mixed lineage leukemia 1 (MLL1), are the most essential contributing vital factors to CSC generation and maintenance (72–74). Increasing published evidences has supported and proved that HIF-1α is the central regulator of induction and maintenance of self-renewal and anti-apoptotic phenotypic properties of various CSCs such as octamer-binding transcription factor 4 (OCT4), SRY (sex determining region Y)-box 2 (SOX2), NANOG (encodes an NK2-family homebox transcripton factor), and Krüppel-like factor 4 (KLF4) (35, 75, 76). Published reports strongly suggests that HIF-1α directly binds to the promoter region of CD24 and induces CD24 overexpression which further accelerates tumor formation and metastasis (77, 78). Additionally, the direct binding of HIF-1α to CD47 and CD133 activate several gene transcription factors that inhibit the phagocytic activity of macrophages and promote the production of CD133+, respectively, which maintain the OCT4 and SOX2-mediated CSC pool of TNBC (79). However, there is controversial evidence in gastrointestinal cancer cells where hypoxia-induced HIF-1α expression decreases CD133 expression. Still, during normoxic states, inhibition of mTOR signaling in gastrointestinal cancer cells reduces the HIF-1α expression that overexpresses CD133 (79).

Moreover, increasing evidence also suggests that HIF-1 transactivates the RNA demethylase ALKBH5 to encode N6-methyladenosine demethylase and increases the stability of NANOG mRNA in BC (80, 81). Additionally, HIF-1 also induced A2BR and activates protein kinase C to transcribe IL-6, IL8, and NANOG, which further promotes stemness, as Lan et al. reported (82). HIF-1α also regulates the 4-trimethylaminobutyraldehyde dehydrogenase, which is associated with cancer cells metastasis, self-renewal, and resistance in BC (80, 83). In turn, HIF-1α expression is also regulated by aldehyde dehydrogenase 1A1(ALDH1A1) *via* retinoic acid signaling in TNBC (83). Studies also suggested that HIF-1α induces the JAK/STAT3 signaling pathway, which can upregulate IL-6 and NANOG while promoting the production of VEGF, responsible for the self-renewal ability and maintenance of the CSC phenotype (81, 84). Another study reported by Lee et al., showed that the production of reactive oxygen species (ROS) in TNBC *via* amplification of MYC and MCL1 overexpress the HIF-1α expression and promotes stemness and chemoresistance in TNBC (85). Crowder et al. discussed the correlation between the antioxidative enzyme superoxide dismutase 2 (SOD2) expression and the expansion of BCSCs in hypoxic conditions. They suggested that TNBCSCs might resist radiation *via* a SOD2-mediated mechanism (86). The tumor microenvironment pH is also associated with CSC’s survival in various cancer types, including TNBC. Interestingly, the pH of TME is tightly regulated by the hypoxia-inducible protein carbonic anhydrase IX (CAIX) by improving the acids transport within the tumor, further increasing the BCSCs survival, expansion and tumor invasiveness (87, 88). Published reports suggest that hypoxia upregulates the CAIX, further enhancing their downstream mTORC1 signaling pathway responsible for regulating triple negative breast cancer stem cells (TNBCSCs) stemness and EMT genes such as Snail and NOTCH (89).

HIF-1α also regulates the expression of ERs, which is a critical indicator of the hypoxic response of BCSCs. Harrison et al. have shown that higher expression of estrogen receptors (ER) is also regulated by HIF-1α and activates the hypoxic responsive factor for the maintenance and proliferation of BCSCs and stimulates the upregulation of Notch genes (90). An interesting study was conducted by Xing et al. group to analyze the expression pattern of Notch ligands in BC patients. They revealed that the expression of Notch and Jagged2 is significantly upregulated in the hypoxic breast tumors, which suggests that they might also regulate the TNBCs maintenance and proliferation and provide critical evidence that Notch and Jagged2 should act as a potential prognostic marker for future clinical applications (91).

**Figure 2:**
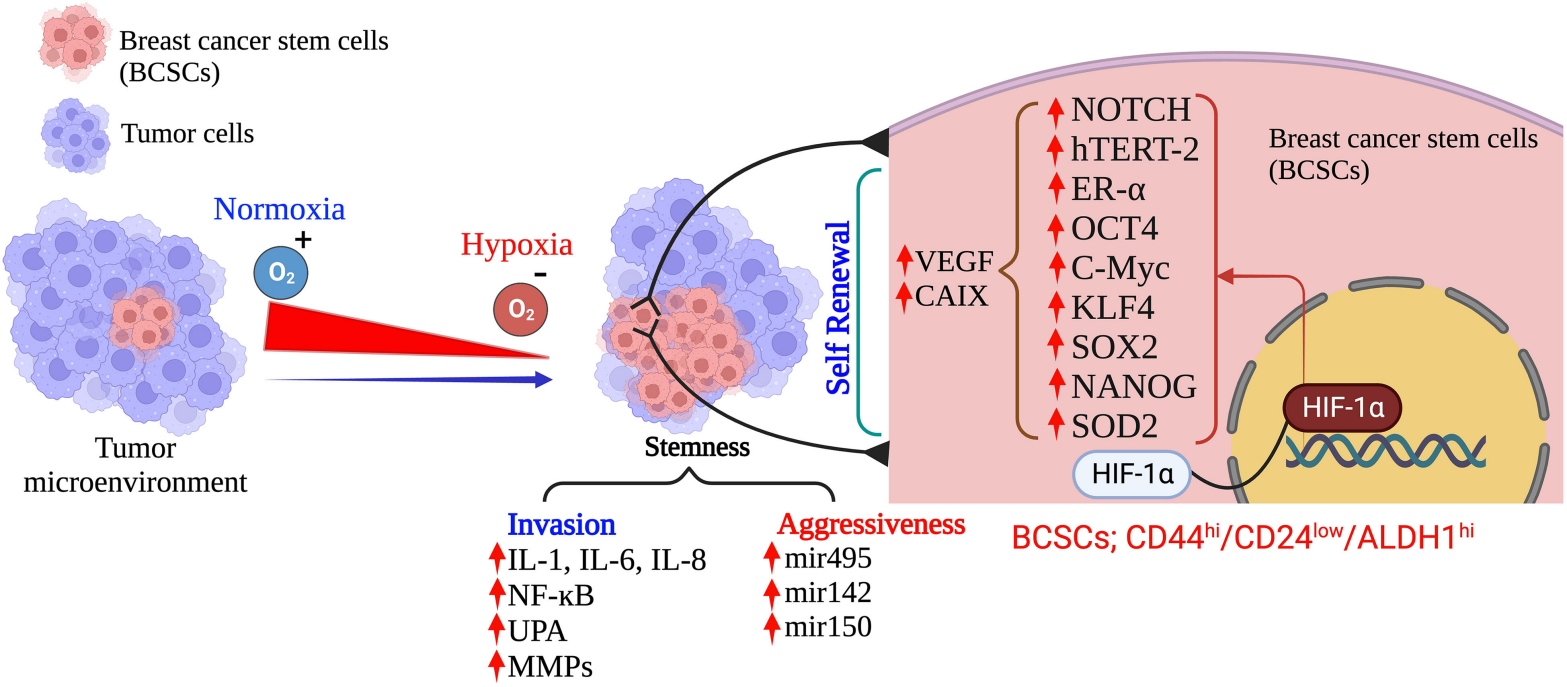
Hypoxia: Determinant of the fate of BCSCs in TNBC. (Created via BioRender.com).

Recent advances in BC research have also shown that hypoxia induces the involvement of microRNAs (miRNAs) in regulating the response of BCSCs (35, 92, 93). Hwang-Verslues et al. reported for the first time that miRNA-495 increases the colony-formation ability, invasive capacity and tumor formation capacity, which further regulates the tumor aggressiveness and hypoxic response of BCSCs (94). Several studies have shown that HIF-1α upregulation in TNBC controls cancer metastasis, CSC self-renewal, and invasion. As a result, the data imply that HIF-1α acts as a direct or indirect upstream regulator of BCSCs in TNBC under low oxygen conditions. This suggests that HIF-1α could be a new target for removing CSCs, which would enhance therapeutic approaches (**Figure 2**).

### 2.3 Hypoxia induces immune escape in TNBC

Tumor immune escape allows tumor cells to survive and grow after evading the host immune system by several mechanisms. It has been reported that hypoxia may induce immunogenic cell death (ICD) within a tumor (95). Earlier investigations have shown that immune escape caused by hypoxia has poor prognostic results and have highlighted the hypoxia pathways as prospective therapeutic targets. Hypoxia signaling has intricate and contradictory functions in triggering immunological escape, encouraging tumor growth and the potential for metastasis, as well as boosting some immunogenic aspects of the tumor microenvironment. Under hypoxic conditions, tumor immune escape involves HIF-1α overexpression (96). HIF-1α inhibits immune cells tumor killing function, which is mediated by regulatory cytokines, granulocyte macrophage colony-stimulating factor (GM-CSF), colony stimulating factor-1 (CSF-1), transforming growth factor-β (TGF-β), CC-chemokine ligand 5 (CCL5), and VEGF in TNBC. In addition, the transcription factor Foxp3 and immune checkpoint molecules like PD-1 also participate in the tumor immune escape mechanism through activation and infiltration of immunosuppressive cells (97).

Myeloid-derived suppressor cells (MDSCs) in TNBC can control tumor-killing cells and immunosuppressive cells by secreting cytokines and suppressing anti-tumor immunity. HIF-1 drives immune evasion and encourages MDSC recruitment. There isn’t much research in TNBC on HIF-1’s modulation of myeloid suppressor cells. HIF-1 controls the communication between TNBC cells and MSCs, leading to the control of MDSCs recruitment. MSCs create CCL5 and bind to CCR5 on TNBC cells in a mouse model (98). The CSF1 receptor on MSCs is simultaneously bound by the cytokine CSF1 produced by basal cells (49). To encourage the recruitment of MDSCs, HIF-1 increases CSF1 and CCL5 signaling.

**Figure 3:**
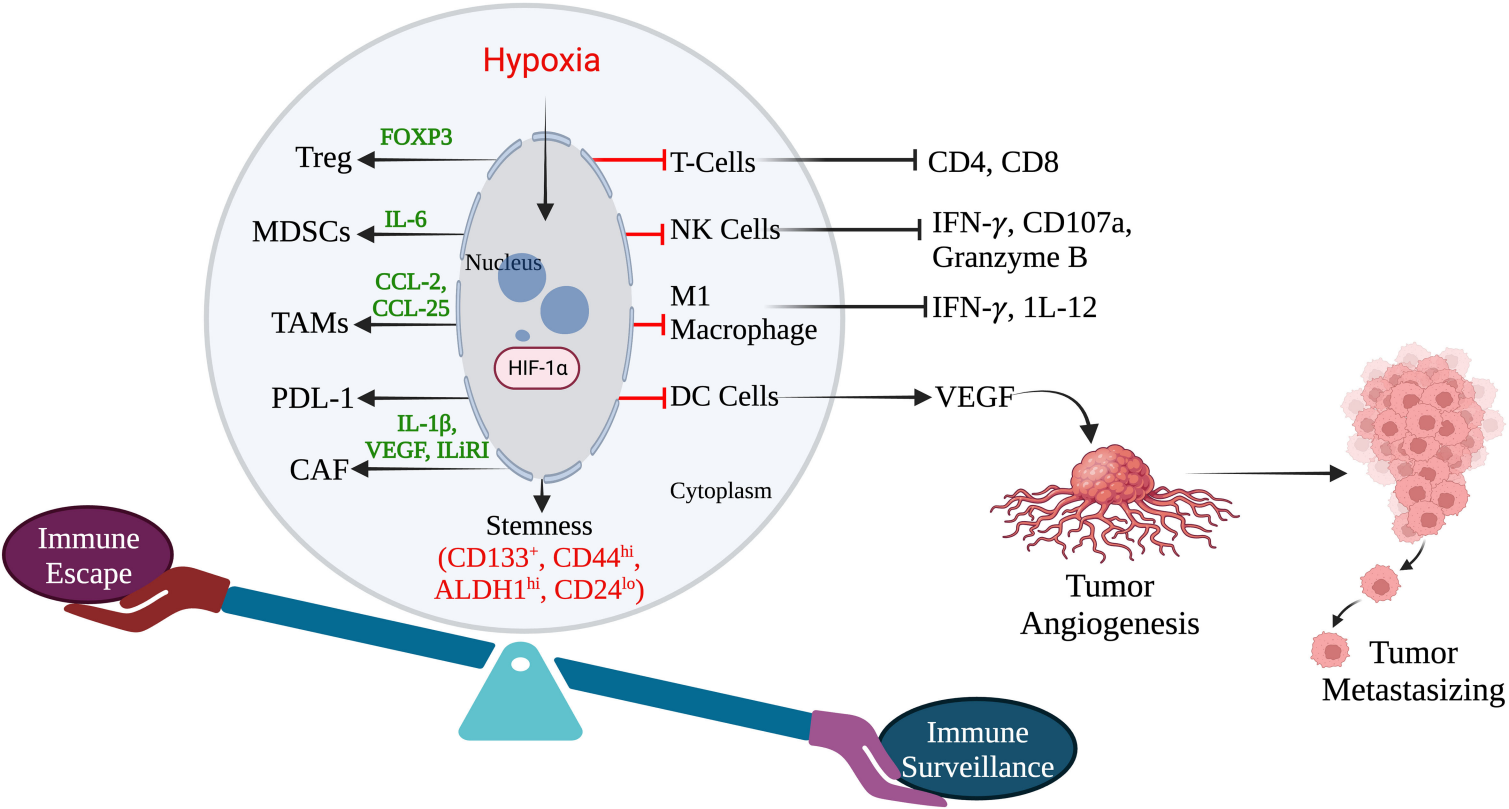
Hypoxia induces immune escape in TNBC. (Created via BioRender.com).

TNBC has higher levels of TAMs, which are strongly linked to a poor prognosis. By producing immunosuppressive molecules, including IL-10 and TGF-β, M2 macrophages have an immunosuppressive effect (99). HIF-1 drives the development of an immunosuppressive milieu and stimulates the polarization of TAMs towards the M2 phenotype (100). Granulocyte-monocyte stimulating factor plays a variety of activities in TAMs, according to prior research. A high concentration of GM-CSF has an immunosuppressive impact by enriching M2 macrophages, whereas a low concentration has an anti-tumor effect by stimulating dendritic cells (DCs) (101). High levels of GM-CSF are generated in TNBC cells under the control of HIF-1 and NF-κB, attracting additional macrophages, and polarizing them into M2-type macrophages (102). Macrophage CSF1, which is secreted by MDA-MB-231 TNBC cells and binds to its receptor on mesenchymal stem cells (MSCs), aids in the attraction of TAMs and MDSCs. Through preserving CCL5/chemokine receptor type 5 (CCR5) communication between MSCs and MDA-MB-231 TNBC cells, HIF-1 controls the expression of CSF-1 (52). As a result, HIF-1 can promote the polarisation of TAMs to the M2 type *via* regulating GM-CSF and CSF1 in TNBC. The T-regulatory (Tregs) cell transcription factor Foxp3 is essential. HIF-1 can regulate the aggregation of immunosuppressive Tregs in TNBC *via* regulating forkhead box P3 (FoxP3) and the C-X-C motif chemokine receptor 4 (CXCR4). *Via* co-regulatory proteins like co-stimulators, transcription factors, co-repressors, and chromatin remodelers, Foxp3 controls the restrictive activity of Tregs (103). CXCR4 is widely expressed on the Treg cell surface and regulates the recruitment of Tregs (104). In TNBC, HIF-1 directs downstream Foxp3 expression by binding to HREs while indirectly enhancing CXCR4 expression by acting on regulatory regions upstream of the CXCR4 transcription start site (105). In patients with TNBC, enrichment of CD8+T is directly linked with improved clinical prognosis and a higher immunological response because CD8+T cells are essential anti-tumor immune cells (106). The tumor-killing ability of HIF-1 in CD8+ T cells is controversial because HIF-1 overexpression in CD8+ T cells increases the level of infiltration and tumor-killing ability of CD8+ T cells (101). The dysfunction of CD8+ T cells was caused by HIF-1’s suppression of immunological effector gene expression under hypoxic settings through histone deacetylase (HDAC-1) and polycomb repressive complex 2 (PCR2)-mediated histone alterations (102). Moreover, under the control of HIF-1, tumor cells produce more adenosine in a hypoxic microenvironment. Adenosine inhibits T cell proliferation and toxicity, promotes T cell death, and inhibits anti-tumor immunological function *via* interacting with adenosine A2A receptors (103). In summary, HIF-1 inhibits CD8+ T cell activation and immune infiltration in TNBC while largely promoting immunological escape. By generating the overexpression of VEGF and PD-1 and encouraging the release of adenosine from tumor cells, HIF-1 regulates the epigenetic mechanism of immune effector genes. This reduces infiltration and impairs CD8+ T lymphocytes capacity to attack tumors (**Figure 3**).

### 2.4 Hypoxia in relation with epigenetic vulnerability in TNBC

Accumulating evidence has also demonstrated that hypoxia-mediated increased expression of HIF is closely linked with manipulating epigenetic plasticity in the tumor cellular system and subsequently induces immune dysfunction (104, 105). Recently, transcriptional and epigenomic analyses by Cong et al. group have shown that Bromodomain-containing protein 4 (BRD4) is the epigenetic regulator. Its dysfunction is critical in mediating several transcription factors by HIF-1α in hypoxic conditions. The study also proved a close association of BRD4 dysfunction with the malignant progression of various tumors; thus, it could be a promising target for different cancer treatments (107). They have also shown that selective degradation of BRD4 subsequently down-regulates the expression of CAIX, a crucial hypoxia-mediated pH gene regulator. Further, it has been proved that overexpression of CAIX induces the acidic environment in various tumors, including TNBC, to adapt the hypoxic environment. Hypoxia-mediated CAIX overexpression also causes a marked reduction in VEGF levels, a master regulator of angiogenesis (107). Another study by Ma et al. demonstrated that hypoxia induces chromatin remodeling in TNBC by the interaction between HIF-1α with HDAC1 and the concurrent PRC2. This interaction epigenetically suppresses the effector gene’s function, which subsequently impacts immune homeostasis and disturbs immune tolerance (102). Chromatin immunoprecipitation (ChIP) assay data demonstrated that hypoxia induces the enrichment of HDAC1 and PRC2. However, it does not change in HDAC 2 and HDAC3 at the effector gene IFN- γ and TNF promoters in the T cells and NK cells (102). Some data suggest that enrichment of HDAC1 and PRC2 by HIF1α-mediated pathways suppresses the functionality of various effector immune cells. Therefore, targeting these pathways through either genetic depletion or therapeutic intervention may be a revolutionary strategy to overcome alteration in immune cell homeostasis. In addition, it might inhibit the disturbance of immune tolerance in TNBC and enhance checkpoint immunotherapy responses. Further, several studies have shown that bromodomain and extra-terminal domain (BET) protein reads histone-acetylation and recruits various transcription factors in TNBC to adapt to hypoxic conditions (108, 109). Ongoing investigations have also shown that hypoxia increases H4ac and H3K27ac, which are associated with transcription and BET protein binding in the HIF targets such as CA9, VEGFA, and LOX promoters (107, 110, 111). Luo et al. discussed and demonstrated selective and specific interactions of HIF-1α with histone demethylase jumonji domain-containing protein 2C but not with HIF-2α (112). By this selective interaction, HIF-1α recruits JMJD2C to the hypoxia response elements of HIF-1 target genes, further reducing the histone H3 trimethylation at lysine 9, and enhancing HIF-1 binding to hypoxia response elements. The enhancement of HIF-1 binding to hypoxia response elements activates the transcription of PDK1, L1CAM, GLUT1, LOX, LOXL2, and LDHA mRNA in human BC biopsies (112, 113). Lambert et al. suggested that lysine demethylases 4C (KDM4C) encoded by JMJD2C interact with HIF1*α* and are involved in metabolic remodeling and metastasis (114). Overall, based on emerging research, epigenetic plasticity plays a critical role in stimulating HIF-1, which further mediates the transactivation of genes that code for proteins implicated in immunological and metabolic tolerance reprogramming in TNBC.

### 2.5 Hypoxia and DNA repair defects in TNBC

Hypoxic tumor microenvironment down-regulates or deregulates the DNA repair pathways by inducing modifications in several transcriptional, translational, post-translational and epigenetic mechanisms (115). This down-regulation or deregulation causes defects in DNA repair pathways linked to extensive genomic rearrangements with a high rate of mutation burden, DNA hyper-replication stress, fragile site induction, and microsatellite instability (MSI). These DNA repair anomalies further lead to the tumor becoming more aggressive and are associated with a significantly worse prognosis/survival in TNBC (116). Therefore to protect the cell against hypoxia-induced replication stress and DNA damage, three primary DNA damage response (DDR) kinases, which include DNA-dependent protein kinase, ataxia telangiectasia and Rad3-related (ATR) protein, and ataxia-telangiectasia-mutated (ATM) kinases are responsible and becomes activated through post-translational modifications (117). Studies have demonstrated that genetic or chemical depletion of ATM or Chk2 in tumor cells has reduced clonogenic survival and increased apoptosis after exposure to hypoxia (118–120). Additionally, hypoxia also activates the ATR/Chk1 response, which subsequently causes pan-nuclear induction of phosphorylation of H2AX (γH2AX) and p53 (121, 122). Further, an emerging study has also demonstrated that depletion of ATR/Chk1 followed by exposure to hypoxia or re-oxygenation significantly reduces cell survival by increasing apoptosis. The study has also unraveled that DNA-PK is activated in hypoxic cells and phosphorylates at Ser2056 of the catalytic subunit that regulates HIF-1 expression (123). The study has demonstrated that ER, PR, and HER2 deficiency in TNBC leads to ATM response hyperactivates (124). Hyperactivation of ATM is predominantly associated with high invasiveness and metastasis of TNBCs by inducing the expression of EMT markers such as Snail and vimentin and reducing the expression of E-cadherin and cytokeratin, which exclusively characterized the epithelial cells (124). Another interesting study demonstrated that hypoxia induces the activation of oxidized ATM, which is independent of DNA damage-mediated ATM activation in TNBCs. Hypoxia-dependent activation of oxidized ATM accumulates the citrate in the cytoplasm. This extracellular accumulation of citrate stimulates the signaling pathway to activate the AKT/ERK/MMP2/9 crucial signaling axis for cell growth, survival, motility and metabolism in TNBC. These findings unravel that oxidized ATM is significantly responsible for TNBC hyperproliferation, invasion and metastasis (125). In addition, available reports have also shown a significant overexpression of ATR and CHK1 in TNBC tissues and promoted tumor progression (126). Meyer et al. demonstrated that an ATR/CHK1 mediated-DDR response prevents the replication stress and induces the resistance of homologous recombination-deficient (HRD) TNBC to mitomycin C (127). This study also suggests that ATR/Chk1 DDR might be a primary mechanism that induces chemoresistance in HR-deficient TNBC (127). Emerging studies have also observed aberrant genetic alteration in other DDR pathways, such as a high prevalence of p53 functional insufficiency and BRCA1/2 mutations (126, 128, 129).

Moreover, HRD is a crucial clinicopathological feature of the BRCA1/2 mutated TNBC, and several studies have proven that specific ATR inhibitors are highly efficient in sensitizing HR-proficient as well as HR-deficient TNBC cells against radiotherapy and inhibit the TNBC proliferation (130, 131). Rad51 is an essential protein for HR that triggers the initiation of the HR process at the sites of DNA damage to repair the damaged DNA. Studies have demonstrated that the knockdown of Rad51 or treatment with a Rad51 inhibitor can enhance the sensitivity to proton therapy and induce proton-mediated clonogenic cell death in TNBC cells (132). Several investigations also revealed that complement 1q binding protein (C1QBP) is highly expressed in hypoxic TNBC and promotes the progression of TNBC. In addition, hypoxia mediated high expression of C1QBP, stabilizes the MRE11 protein (DDR protein) in MRN complex (MRE11/RAD50/NBS1) and inhibiting MRE11 exonuclease activity which makes TNBC resistance to chemotherapy (133). Study has also demonstrated that blocking RAD50 within the MRN complex sensitizes CSCs and chemo-resistant BT-549 and MDA-MB-231 TNBC cell lines to chemotherapeutic drugs (3, 134). Recent studies have also shown that BRCA1 is critical in resolving DNA double-strand breaks (DSB) by HR repair, particularly DSB associated with cross-links at the end of DNA replication forks. Moreover, available reports suggest that mutation or deficiency of BRCA1 genes altered the HR-related gene, which further leads to HR deficiency in TNBC, makes TNBC more sensitive to the specific therapies that generate cross-links or DSBs fragments, including platinum drugs, alkylating agents, anthracyclines and PARP inhibitors (134, 135).

Non-homologous end joining (NHEJ) repair pathway is another crucial pathway that recognizes DSBs *via* Ku70/80 heterodimer in association with DNA-PKcs to repair damaged DNA in various cancer cell normal cells (136). Although it’s still debated, the exact effects of hypoxia on NHEJ still need to be explored. It is most likely that several hypoxia-mediated molecular mechanisms altered the crucial NHEJ protein expression and deregulated or downregulated functionality of NHEJ-associated proteins. Studies have demonstrated that long noncoding RNA (lncRNA) overexpressed in TNBC and hyperactivated the NHEJ pathway by supporting Ku80 and DNA-PKcs to repair of DSBs and promote tumorigenesis (137). Interestingly, the hypoxia-mediated amplification of EGFR activity and P53 mutation in TNBC is also responsible for the high expression of lncRNA in non-homologous end-joining pathway 1 (LINP1) in TNBC (137). Zhang et al. demonstrated by RNA-immunoprecipitation assays (RNA-IP) that the association between LINP1 and Ku80 or DNA-PKcs induced by ionizing radiation (IR) exposure to tumor cells. However, blocking LINP1 radio sensitizes the TNBC tumor for radiotherapy (137).

Hypoxia also alters the mismatch repair (MMR) pathway by mutating the major MMR proteins MLH1 and MSH2, including MSH6 (138, 139). Severe hypoxic conditions downregulate MLH1 and MSH2 in a HIF-independent manner. However, in moderate hypoxic conditions, MSH2 and MSH6 downregulate in HIF and P53-dependent manner (138, 140). Although it’s unclear, available reports suggest that hypoxia-mediated MMR downregulation also requires HDAC activity (141). These mutations or deregulation further led to microsatellite instability and are characterized by decreased or increased repeated nucleotide sequences (138–140).

Moreover, a high incidence of MSI observes in various tumor models, such as colorectal, ovarian, stomach, urothelial, central nervous system, and adrenal gland. The high incidence of MSI is further responsible for developing several malignant mutations and tumor evasion. However, based in published reports so far, only limited data are available on disease prevalence, and the prognostic significance of MMR-d/MSI-H in BC. Additionally, in TNBC, a low incidence rate of MMR-D/MSI-H is observed. Recent clinicopathological studies conducted in 440 patients with TNBC demonstrated no correlation between MMR-d/MSI-H and clinicopathological parameters such as PD1/PDL-l immune checkpoint expression and survival. Another study also revealed that low expression MMR-D/MSI-H characteristics in TNBC may not be a practical predictive marker for immunotherapy by using immune checkpoint inhibitors of PD1/PDL-l (139).

Emerging studies also demonstrated the hypoxia mediated reduction of several base excision repair (BER) factors expression such as OGG1, MYH, POLB, APE1, RPA, PCNA and ASCIZ/ATMIN in TNBC which leads the TNBC BER-deficient (BER-d). Moreover, induction of oxidative or alkylating glycosylation *via* low protein production in TNBC is significantly associated with oxidative or alkylating glycosylases through low protein production (142, 143). The available report suggests that a combination of PARP inhibitors that directly impact BER signaling and other relevant therapies that generate ROS and induce defective glycosylation would be more precise and targeted therapies for TNBC treatment (142, 144).

The effects of hypoxia on nucleotide excision repair (NER) remain debatable. Several NER gene expressions, such as XPA, XPB, XPD and XPG, do not change after hypoxia exposure (115). However, some evidence indicates that severe hypoxia suppresses the NER capacity and demonstrates hypermutability to UV irradiation (145). Another study conducted in HCC1806 and MDA-468 TNBC cells showed the inactivation of major NER proteins such as ERCC1, XPA, and XPF (146). These proteins play an essential role in recognizing and excision bulky base lesions. The inactivation of scaffold protein, XPA, induces severe sensitivity to UV radiation and a high risk of carcinogenesis (147). These studies suggest that the induction of deregulation of NER in TNBC could offer revolutionary new targets for the treatment of TNBC either alone or in combination with other therapies. In summary, understanding of DNA repair defects in TNBC can potentially be used to overcome resistance to treatment.

## 3 TNBC and treatment strategies

**Figure 4:**
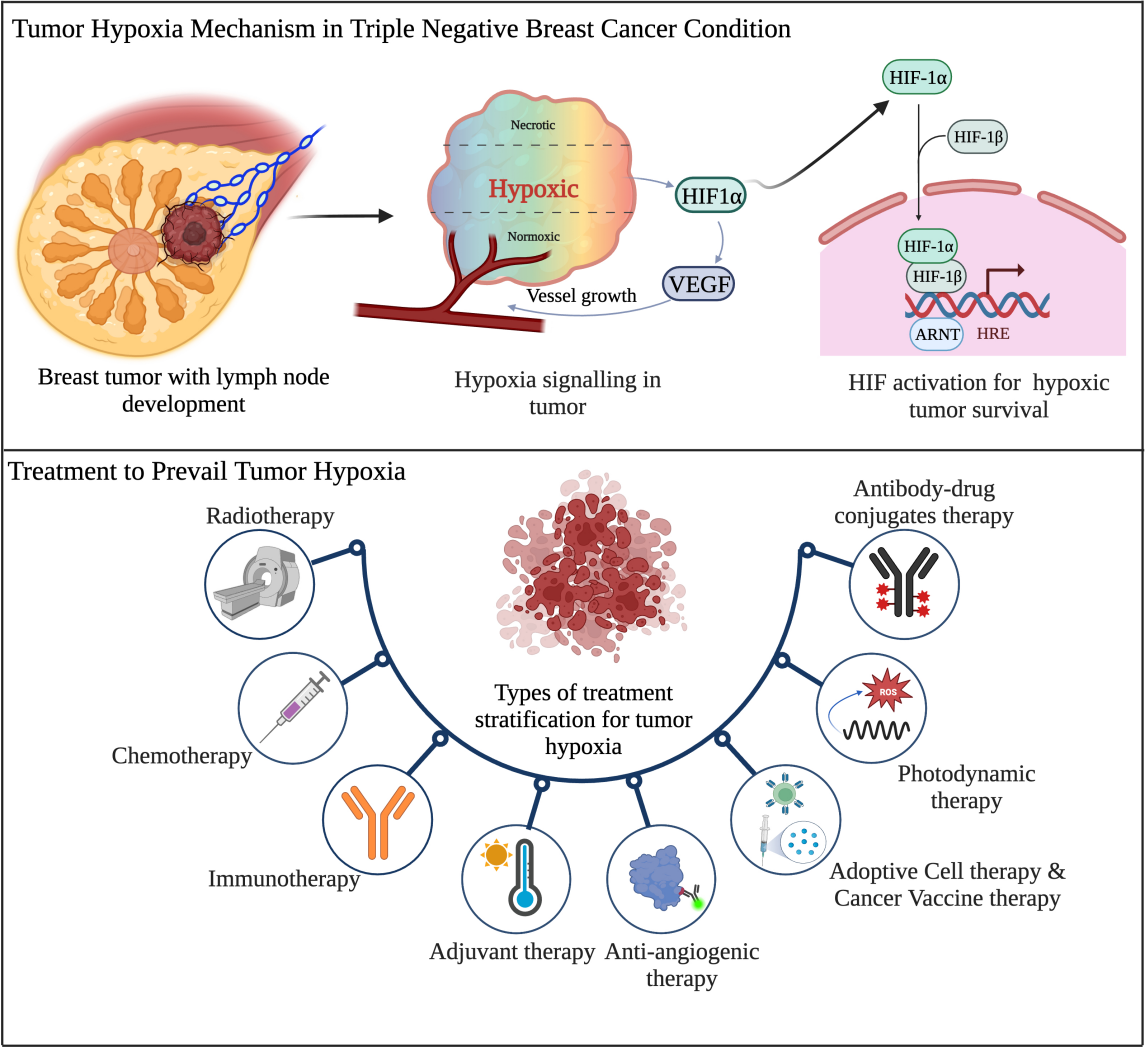
Schematic overview of tumor hypoxia mechanism and existing therapeutic regimens in TNBC. (Created via BioRender.com).

TNBC harbors a highly heterogenous and aggressive behavior with a distinct metastatic pattern. It also contains a high mutational burden and activates various tumor initiation signaling pathways. TNBC is the highest malignant BC subtype with a poor clinical outcome. Therefore, the current treatment options are limited to surgery, chemotherapy, and radiotherapy. The limited clinical ramifications of TNBC have lagged other types of BC. Due to its hyper-progression and aggressiveness, it remains challenging BC to treat and minimal options are available for treating this form of BC. Many efficacious treatments for most BC are limited to inhibiting the growth-stimulating effects of PR, ER, and HER2. Finding novel and potent therapies for TNBC remains a crucial clinical need because it lacks these growth-stimulating receptors. To develop specific efficacious drugs, new experimental approaches need to be adequately investigated in pre-clinical and followed by clinical trials platform on patients diagnosed with TNBC. **Figure 4** summarizes the tumor hypoxia mechanism and current treatment approaches for TNBC. 

### 3.1 Radiotherapy

In 1920, Hall et al. showed that the tissue inadequately supplied with oxygen was highly resistant to ionizing radiation (IR) (148, 149). In 1953, Gray’s seminal paper demonstrated that the preclinical study reported that decreasing hypoxia enhanced oxygen distribution and increased radio sensitivity. However, these findings facilitate the way for several studies summarizing the mechanism of radio resistance induced by hypoxia. On the whole, HIF-1 and radiotherapy have a complicated, two-sided relationship.

Following radiotherapy, which induces hypoxia-dependent HIF-1 expression in the tumor, causes vasculature damage and a consequent oxygen level deficit. Earlier, Moller et al. demonstrated that following IR, the murine tumor model showed increased HIF-1 expression. As a result, the irradiated mouse tumors gave the impression of radiation-induced reoxygenation and displayed relatively high oxygenation levels in the tissue (150). Authors found two unexpected results were found in the same study: (a) ROS activation led to HIF-1 stabilization, and (b) the increased translation of HIF-1 transcripts owing to reoxygenation caused the breakdown of monomers referred to as “stress granules". Previous studies endorsed ROS-directed HIF-1 stabilization, most likely by decreasing PHD enzyme activity (151, 152). However, another hypothesis emerged as P13/AKT/mTOR pathways, which result in increased HIF-1α expression, and Ras/Raf/ERK/MEK pathways, which always attribute ROS-mediated regulation of HIF-1 expression.

Additionally, the stabilization of the HIF-1 protein during IR is linked to heat shock protein 90 (Hsp90) (153, 154). In response to IR, endothelial cells underwent a stress reaction that resulted in either recovery or function loss and cell death. The critical destiny is determined by a number of variables, such as intrinsic TME characteristics, fractionation schedule, and total dose (155). VEGF is at the centre of this interaction as an HIF-1 target gene and a crucial regulator of tumor vascularization (156). Radiation-induced fibrosis, or RIF, is a dose-limiting postradiotherapy consequence. In reaction to radiation exposure, the body undergoes an aberrant wound-healing process (RIF), which leads to a self-replicating fibroproliferative condition (157). IR-induced DNA damage triggers an initial inflammatory response, which is followed by endothelial cell failure and hypoxia. This causes aberrant collagen and other extracellular matrix protein buildup as well as an atypical activation of fibroblasts (sometimes referred to as the activated state of myofibroblasts). TGF- is an important participant in this process, and one of the critical RIF-initiating events is ROS-mediated post-translational activation of TGF- β (158).

One of the most notable instances of how HIF-1-mediated metabolic reprogramming might directly counteract radio-resistance in relation to radiation is glycolysis-induced activation of the pentose phosphate pathway (PPP). PPP activation decreases oxidized glutathione, restores nicotinamide adenine dinucleotide phosphate (NADPH), and shields cancer cells from ROS (159). In a recent clinical trial looking at first-time tumor hypoxia in SBRT, Song et al. first showed a clinical study investigating tumor hypoxia in SBRT with high doses of single fraction radiation delivered to patients with lung cancer (160). Overall, there is still much to learn about the importance of tumor hypoxia in the era of hypofractionation therapy. Stereotactic body radiation (SBRT) replaced traditional fractionated radiotherapy techniques, and this change sparked a new quest for hypoxia-modifying radiotherapy drugs. Image-guided radiation, intensity-modulated radiotherapy, volumetric modulated arc treatment, targeted combinatorial drug therapies, and immunotherapy are examples of contemporary innovations in radiotherapy delivery and imaging approaches that have improved radiotherapy’s therapeutic index (161, 162).

The conventional radiotherapy involves the usage of either external beam, like that of a regular x-ray or more feasible internal radiation known as brachytherapy, in which a sealed radiation source is inserted to target the cancerous area. However, Smith et al. demonstrated that a high increased risk of subsequent mastectomy after brachytherapy treatment compared with external beam therapy (163). Proton beam therapy (PBT) has a lower administrative dose than conventional radiotherapy and allows the majority of the radiation dose to be explicitly focused on the tumor, is also more frequently used. This can reduce the needless irradiation of nearby normal tissues, which will lessen the likelihood of side effects. Emerging studies demonstrated a dosimetric comparison between brachytherapy and PBT and intensity modulated proton radiotherapy technique (IMPT) and suggested a comparability similar dose effect in BC patients and also suggested to use of PBT for significant outcome (164) (**Table 1**). Modern developments in clinical radiotherapy technology are aimed at enhancing the capabilities of the radiotherapy machines and altering the local mode of radiotherapy to maximize the accuracy of irradiating tumor tissue while minimizing damage to healthy tissue.

**Table 1:** Clinical trials evaluating different therapeutic approaches in patients with TNBC.

S. No.	Type		Treatment	TNBC patient population	Mechanism of Action	Clinical Phase	Status	References
1.	CHEMOTHERAPY		Paclitaxel	52	p53/p21 pathway or Raf-1 kinase activation pathway	II	Completed	(165)
2.			Nab-paclitaxel	903	Inhibit of tumor growth	II/III	Completed	(165, 166)
3.			Docetaxel	127	Attenuate the effect of BCL-2 and BCL-XL gene	II/III	Completed	(167)
4.			Tesetaxel	674	PDL-1 Inhibitor	III	Completed	(168)
5.			Doxorubicin	52	Blocking the topoisomerase 2	1/1b	Completed	(169, 170)
6.			Epirubicin	53	Inhibit totpisomerase II activity	II	Completed	(171)
7.			Pegylated liposomal doxorubicin	39/113	Blocking the topoisomerase 2	1/III	Completed	(172, 173)
8.			Cyclophosphamide	40	Inhibiting humoral 1 and 2	II	Completed	(174)
9.			Cisplatin	47	Inhibit the DNA synthesis	II	Completed	(174, 175)
10.			Carboplatin	647	Inhibit the DNA synthesis	II	Completed	(176)
11.			Eribulin mesylate	762	Reversing epithelial-mesenchymal transition to mesenchyal-epethilial transition.	III	Completed	(177)
12.			Capecitabine	434	Inhibit thymidine monophopshate (ThMP) synthesis	II/III	Recruiting	(178)
13.			Gemcitabine	50	Activates p38 MAP kinase pathway	II	Completed	NCT02435680; Novartis (Novartis Pharmaceuticals), 2021
14.			Fluorouracil	647	Inhibit thymidylate synthesis (TS)	III	Completed	NCT01216111; Zhimin Shao, Fudan University, 2020
15.			Ixabepilone	91	Microtubule inhibitor, blocks cell growth by stopping cell division.	–	Recruiting	(179)
16.			Taxanes	-	Microtubule inhibitor and inactivate HIF-1α pathway	-	-	(180)
1.	RADIOTHERAPY		External beam radiation therapy (EBRT)	-	Radiation to destroy cancer cells	I	Unknown	(163, 164, 181)
2.			Brachytherapy (149)	-	Limits radiation treatment to the tissue surrounding the lumpectomy	I	Unknown	(163, 164, 181)
3			FLASH-RT	-	Limits the radiation toxicity to the surrounding healthy, normal tissues.	-	-	(182)
4			IMPT	-	Radiotherapy technique to treat tumors in layers of spots at varying depths by altering the number localized proton dose deposition, energy penetration, and magnetic deflection.	-	-	(164)
5			PBT	-	Reduce the needless irradiation of nearby normal tissues and side effects	-	-	(164)
1.	ANTIANGIOGENIC THERAPY		Bevacizumab	54	Angiogenesis agent by Inhibits the VEGF binding to it cell surface receptor	II	Completed	(183)
2.			Lenvatinib	31	Multiple receptor inhibition VEGFR-1, VEGFR-2, VEGFR-3, FGFR1, FGFR-2, FGFR-3, FGFR-4, PDGFRa, RET and c-KIT.	II	Recruiting	(184)
3.			Apatinib	32	Inhibits VEGFR-2 receptor reduce tumor vasculature	I	Completed	(185)
4.			Cabozantinib	35	Inhibits VEGFR-1/2 and -3, TRNKB, FLT-3,KIT,TIE-2, MET,AXL and RET	II	Completed	(186)
5.			Anlotinib	30	Dual signaling blockade VEGFR2 and MET pathways	I	Unknown	(187)
1.	ADJUVANT THERAPY		Accelerated radiotherapy with carbogen and nicotinamide	MDA-MB-231 TNBC xenograft tumors	Radiation with carbogen to destroy cancer cells	0	Preclinical	(188)
2.			Hypothermia	2	Heat tissue high as 113 ^0^ F to kill cancer cells	0	Unknown	(189)
1.	PHOTODYNAMIC THERAPY (PDT)		Protoporphyrin IX	TNBC cell lines (HCC1395, BT-20, MDA-MB-231, and Hs578T)	Inhibits Ras/MEK pathway.	Preclinical	*In-vitro*	(190)
1.	IMMUNOTHERAPY	Immune-checkpoint inhibitors (ICI)	Pembrolizumab	32/84/170	Inhibit PD-1 pathway.	Ib/II/III	Active	(191–193)
2.			Atezolizumab	115/41	Blocking its interaction with PD-1 and B7-1.	I/II	Active	(194)
3.			Avelumab	58	Blocking its interaction with PD-1 and B7-1.	I	Active	(195)
4.			JS001	20	PD-1 Inhibitor	I	Active	(196)
5.			Nivolumab	51	Blocking interaction with PDL1 and PDL2.	I/II	Active, Not Recruiting	(197)
6.			Durvalumab	45	Blocking interaction with PDL1 with PD-1 and CD80.	II	Completed	(198)
1.		T-Cell Targeted Modulators	Anti-CTLA4	35/129	Inhibit CTLA4 and suppress natural killer cell maturation.	I/II	completed	(199)
2.			Anti-LAG3	363	Inhibit LAG3	II	Completed	(200, 201)
3.			Anti-TIGIT	-	Inhibit TIGIT	0	Not yet	(202)
4.			Anti-CD137 agonistic antibody	-	Suppress CD137 receptors.	0	Not yet	(203)
5.			Anti-OX40 agonistic antibody	-	Suppress OX40 receptors	0	Not yet	(203)
6.			Anti-CD40agonistic antibody	-	Suppress CD40 receptors	0	Not yet	(203)
1.		Immunomodulators	Acetylsalicylic acid	-	Disrupts NFkappaB-IL6 signaling axis and inhibits cyclooxygenase (COX) enzyme.	-	Not yet	(204)
2.			COX2 inhibitors (indomethacin)	-	Disrupts cancer-cell fibroblast signaling.	-	Not yet	(205)
3.			Recombinant IFN-alpha-2b activating. TLR3 receptors	-	Stimulates JAK-STAT pathway.	-	Not yet	(206)
4.			A2AR antagonists	-	Decreases the immunosuppressive mechanisms such as Tregs, CTLA-4, TGF-beta and COX2, eicosanoid mediators.	-	Not yet	(207)
5.			CSF-1R inhibitors	-	Target M2 macrophages/TAM	-	Not yet	(208)
6.			Anti-TGF-beta antibodies	-	Promotes T cell infiltration.	-	Not yet	(209)
7.			L-NMMA (pan-NOS inhibitor)	15/24	Increases circulating IL-6 and IL-10 cytokines, in contrast, CD15+ neutrophils and decrease in arginase.	I/II	Recruiting	(210)
8.			Oncolytic reoviruses	-	Selectively replicate in cancer cells and then kill them without damaging the healthy cells by enhancing the recruitment of innate immune function and inducing tumor cell apoptosis.	-	Not yet	(211)
9.			Anti-IL1beta antibodies	-	Decreases IL-6 production through a transglutaminase 2/NF-κB pathway.	-	Not yet	(212)
10.			Poly-ICLC	-	Increase cytokines and immune response.	-	Not yet	(213)
11.			Anti-IL-6R antibodies	-	Decreases the breast cancer cell aggressiveness.	-	Not yet	(214)
1.	ADOPTIVE CELL THERAPY (ACT)		EGFR/CD276	30	Induces T cell activation	I	Recruiting	(215)
2.			ROR1-targeted CAR T cell (LYL 797)	54	Harbors synthetic Notch receptors specific for EpCAM or B7-H3 (expressed by ROR1-expressing tumor cells) and reported that these CAR-Ts safely mediated efficient tumoricidal activity without toxicity	I	Recruiting	(215)
3.			NKG2DL-targeting CAR-grafted gamma delta (gd) T cells	10	Secretes cytokines and chemokines and exhibiting cytotoxicity	I	Recruiting	(215)
4.			c-met-RNA CART T cells	6	Reduces the proliferation and migration capacity of TNBC	0	Recruiting	(215)
5.			CART-TnMUC1 cells	16	Target antigen-dependent cytotoxicity and released cytokines, chemokines, and granzyme B	I	Recruiting	(215)
6.			Anti-meso-CAR vector transduced T cells	20		I	Recruiting	(215))
7.			Mesothelin-specific chimeric antigen receptor positive T-cells	186	Activate T-cells.	I	Recruiting	(215)
8.			PD-1+ TILS	20	Inhibition of T cell function and depletion of T cells	i/ii	Recruiting	(102, 215)
9.			TC-510	115	Elicits T cell response through mesothelin.	i/ii	Recruiting	(215)
1.	CANCER VACCINE (CV)		Dendritic cell vaccine	23	Induces the IFN-γ-production by CD4+ T cells.	II	Active/not recruiting	(211)
2.			AE37 peptide therapeutic vaccine	29	Activate CD4+ immune response and stimulate T-helper cells against HER2/Neu expressing cancer cells.	II	Active/not recruiting	(211)
3.			Neoantigen personalized DNA vaccine	18	Induces the number of neoantigen-specific cytotoxic T cells.	I	Recruiting	(211)
4.			PVX-410	20	Induces cytotoxic T lymphocytes (CTLs) to target specific tumor associated antigens such as highly over-expressed tumor antigens XBP1, CD138 and CS1.	II	Recruiting	(211)
5.			GP2	456	Activate CD8+ response against the HER2 antigen	II	Completed	(216)
6.			Nelipepimut-S	275	Stimulate cytotoxic T lymphocytes to lyse of HER2-expressing cancer cells.	II	Completed	(216)
7.			Tecemotide	400	Stimulate an antigen-specific cellular immune response against MUC1+ cancer cells.	II	Completed	(216)
8.			AS/OBI-821	349	Reduces the Tregs, therefore increases the humoral response.	II	Completed	(216)
9.			H/K-HELP	12	Increases the IFN-γ-production by CD4+ T cells and induces Th1 dependent induces cellular and humoral immune responses.	I	Completed	(216)
10			P10s-PADRE	24	Induces the expression of CD16, NKp46 and CD94 expression on NK cells and a serum content of IFN-γ produced by CD4+ T cells.	1/II	Recruiting	(211)
11.			Galinpepimut-S	90	Stimulate CD8+ and CD4+ T-cell responses.	II	Recruiting	(211)
12.			KRM-19	14	Stimulate cytotoxic T lymphocytes and induces the IFN-γ-production by CD4+ T cells.	II	Completed	(216)
13.			Tumor lysate-pulsed DC 23 vaccine	29/21	Induces the IFN-γ-production by CD4+ T cells.	II	Completed	(216)
14.			RO7198457 (iNEST)	272	Enhances anti-tumor activity of atezolizumab (anti–PD-L1) by increasing the number of neoantigen-specific cytotoxic T cells.	II	Active/not recruiting	(211, 217)
15.			NANT cancer vaccine (NCV)	79	Enhances immunogenic cell death by activating the T cell and NK therapy and also reduces the Tregs	I	Active/not recruiting	(216)
16.			Elenagen	27	Reduces in the population of suppressive cells in the TME, including regulatory T-cells (Tregs) or myeloid-derived suppressor cells (MDSCs).	I/II	Completed	(216)
17.			p53MVA	11	Induces the frequencies and persistence of p53-reactive CD8+ T cells.	I	Active/not recruiting	(216)

Ultra-high dose rate (UHDR) radiotherapy called FLASH radiotherapy (FLASH-RT) has been expected as a new method in recent years (**Table 1**). In multiple trials, radiation toxicity to the surrounding healthy, normal tissues was markedly decreased, and tumor growth was suppressed, with tumor control on par with conventional dose rate irradiation. It is generally acknowledged that FLASH irradiation has great future potential and is perhaps the most significant discovery in the history of radiation treatment, despite some researchers’ skepticism over FLASH-effectiveness RT’s in treating cancer patients (182).

### 3.2 Chemotherapy

Chemotherapy aims to weaken the cancer cell defenses against apoptosis, mitotic catastrophe, autophagy, and necrosis in order to promote cancer cell death. Apoptosis and autophagy, to start, are genetically programmed. Second, passive reactions to extreme cellular mistreatment include necrosis and mitotic catastrophe (218). The majority of chemotherapy medicines cause DNA damage. Apoptosis is the main method of cell death in reaction to DNA damage, even if medications that cause DNA damage can also cause necrosis as an alkylating agent or autophagy as an etoposide (219). It is well known that hypoxia lowers the effectiveness of chemotherapy treatments since these drugs need oxygen to act as an electron acceptor in order to kill cells.

Hypoxic tumor cells are discussed in this review as a way to avoid chemotherapy (220). Hypoxia drastically altered the transcription of cells, principally by activating HIF-1. HIF-1 is made up of two subunits: HIF-1/ARNT, which is constitutively stable and HIF-1, which is oxygen-sensitive. Low oxygen levels enable the development of active HIF-1 by preventing post-translational changes of the HIF-1 subunit. Through controlling expression, angiogenesis, autocrine growth factor signaling, invasion, and treatment failure often due to the presence of ABC transporters, HIF-1 contributes to metabolic reprogramming (221, 222). In addition, the HIF-1 subunit helps control p53. Its main function is to control the expression of numerous genes that code for proteins, which helps to control apoptosis. The preservation of genomic integrity depends heavily on the transcription factor p53. An et al. were the first to discover that HIF-1 is essential to the p53 pathway. The stability of the p53 protein under extreme hypoxia conditions was determined by the authors in previous study (223). In addition, they noted that the phosphorylation of HIF-1 has a dual role function in controlling apoptosis. According to Suzuki et al., the dephosphorylated form of HIF-1, which predominates in severe hypoxia and has a greater affinity for ARNT than the phosphorylated version of HIF-1, is necessary for p53 stabilization through HIF-1. On the other hand, research by Pan et al. and others have demonstrated that even intense hypoxia is insufficient to stabilize the p53 gene without the secondary holding provided by rigorous hypoxia, such as food shortage and pH collapse (224, 225). For many years, the only way to completely eradicate tumor cells and prevent their development and proliferation by chemical agents used in cancer therapy was through chemotherapy. Chemotherapy’s main strength and most compelling flaw is its inability to distinguish between cancer cells and healthy cells, which results in severe toxicity and side effects. Cancer treatment has changed significantly during the past 20 years from broad-spectrum cytotoxic medications to tailored treatments (226). Targeted medications now have a higher potency and lower toxicity as compared to traditional chemotherapeutic drugs since they can directly target cancer cells while protecting healthy cells. Targeted medications can be broadly categorised into two groups: (a) small compounds like imatinib, which the US Food and Drug Administration (FDA) licenced for clinical use in 2001 (227); and (b) macromolecules such monoclonal antibodies, polypeptides, nucleic acids, and antibody-drug conjugates (228, 229). It is a well-known medication that will be easily developed and has entered a golden stage of development, which has been supported by the approval of targeted drugs.

Over the last 20 years, there has been a significant increase in targeted FDA-approved medicines for cancer treatment. Small-molecule targeted drugs, on the other hand, have several advantages over macromolecule targeted drugs in terms of cost, pharmacokinetic properties (PK), patient compliance, drug storage, and market availability. In the United States and China, 89 small anticancer molecules have been approved. Small molecules for anticancer drugs face numerous challenges, including drug resistance and a low response rate. Many strategies for administering chemotherapeutic medications can now be used to extend life. Chemotherapeutic drugs are commonly administered in a combinational approach. For various types of cancer, various combinations are available. In this review, we will focus on a few drugs that are commonly used to treat various cancer types, such as doxorubicin, cyclophosphamide, cisplatin, 5-fluorouracil, and others (**Table 1**). In general, chemotherapeutic agents are administered to patients who may be able to withstand the treatment. Because less cell death is observed in tumor masses, current clinical setup chemotherapeutic regimens are used to treat cyclic tumors. As a result, dose reputation is required to reduce tumor size. There are a few drawbacks to the duration and frequency of chemotherapies, which are limited by patient toxicity (230).

### 3.3 Immunotherapy

Hypoxia refers to solid tumors and attributes the selection of intrusive and destructive malignant clones displaying resistance to RT, traditional chemotherapy, or small molecule targeted therapy. The recent clinically applicable immunotherapy-based checkpoint inhibitors (ICPIs) and chimeric antigen receptor (CAR) T- cells, has evidently altered the prognosis for certain tumors (231). Notably, hypoxia triggers the angiogenesis and causes immunosuppression, which is termed another dilemma of hypoxia-induced immune resistance. While these treatment strategies reveal both a promise and a despair in terms of efficacy and safety in phases of clinical trials, they correspond to the future solution to appreciate the efficacy of immunotherapy in contrast to hypoxic and therapy-resistant solid tumors.

However, based on the prediction, tumor hypoxia has shown poor outcomes across all types of cancer. Despite the success of T-cell immune checkpoint blockade in treating melanoma, abrasive adenocarcinomas of the prostate and pancreas are mostly resistant to CTLA-4 and PD-1 antibody treatment in the mice and humans. Previously, Midan et al. reported that hypoxic zones of the tumors endure infiltration by T cells, even in the context of vigorous infiltration of T cells in normoxic regions of the same tumor (4). Beyond the dearth of admissibility to tumor-specific T-cells, hypoxia energizes the foundation of an extremely interdependent network of immunosuppressive stromal cells. Based on the Midan et al. finding it was noted that the critical population of myeloid-derived suppressor cells (MDSCs) and myofibroblasts which act together to suppress T-cell responses and intervene in immunotherapy resistance (4).

Tumor hypoxia primarily affects antitumoral immune activity by inhibiting the native immune system and immune killing mechanisms. Many studies have concentrated on immunosuppressive elements in the tumor microenvironment, including MDSCs, Treg cells, and TAMs in the hypoxic zone of solid tumors (232). Inside the hypoxic TME, HIF-1, a key hypoxia transcriptional factor, controls MDSC activity and differentiation. According to research by Norman et al. on this subject, enhanced HIF-1-dependent arginase activity and nitric oxide production in tumor-dependent MDSCs make them more immunosuppressive than splenic-derived MDSCs (233). Another study discovered evidence that HIF-1 controls PD-L1 expression by directly attaching to components that have hypoxia-responsive properties in the proximal promoter of PD-L1 (234). Atezolizumab is the first FDA approved ICI monoclonal antibody for the treatment of mTNBC which targets PDL-1 and later pembrolizumab is also approved for mTNBC treatment in combination with chemotherapy based on the positive clinical trials result with atezolizumab and pembrolizumab monotherapy in TNBC (215). There are several clinical trial studies are registered on clinicaltrial.gov which implies that using ICI either alone or in combination with other therapy could be a promising strategy in TNBC treatment which is summarizes in **Table 1**. In addition to ICI several other combinations with immunotherapies such as immunomodulators (acetylsalicylic acid, indomethacin, IFN-α2b etc.), T-cell targeted modulators (CART-TnMUC1, TC-510 etc.) are still under investigation and these may contribute to the development of precision immunotherapy for TNBC (215) (**Table 1**).

### 3.4 Adjuvant therapy

The main challenge in overcoming tumor hypoxia in a clinical setting is to increase oxygen delivery. Horsman et al. previously showed that hyperbaric oxygen (HBO) treatment involves breathing 100% oxygen 2-4 times daily at normal atmospheric pressure. The results showed increased saturation of hemoglobin and oxygen levels in the circulation (235–237). In general, HBO treatment is administered during or shortly before radiation therapy. Previously, in the 1970s, Chaplin et al. reported that, when compared to normal air, patients with head and neck squamous cell carcinoma responded better to HBO treatment in terms of local control in a multi-center randomized trial (238).

On the other hand, carbogen breathing produced disparate results, which might be explained by variations in the number of patients who underwent carbogen breathing (239). Although the treatment for high-risk brain stem glioma in pediatric patients was well tolerated, there was no evidence of any benefit when radiation therapy was added. Siemann et al. previously reported that the combination of nicotinamide, a vitamin B3-derived molecule, and radiation appears to target both acute and chronic hypoxia (240). Additional research suggests that nicotinamide reduces ACT hypoxia by sporadically preventing vascular shut-down. Van Laarhoven et al. conducted ARCON trials, and other groups demonstrated improved patient survival, particularly in bladder and laryngeal cancer (241–243). However, the ARCON trials have demonstrated the efficacy of carbogen breathing as an adjuvant therapeutic regimen (244).

Interestingly, one of the adjuvant treatments called hyperthermia (HT) involves heating tissue over physiological temperatures (40–450°C). Although HT can be given directly to tumor masses, it is typically utilized as an adjuvant therapy alongside chemotherapy or radiation therapy due to technical challenges in obtaining cytotoxic temperatures (153, 154). However, in patients, ultrasonography is used to administer HT superficially or intravenously. Microwaves, radio frequencies, and electromagnetic radiation are all examples of electromagnetic radiation. In the 1970s and 80s, HT’s positive impacts were seen in primary and secondary cells in the culture system, as well as in the preclinical evaluation of animal models and patients. Previously, five randomized trials combining radiation and HT exhibited benefits in recurrent melanoma, cervical and BC patients (155–157). HT has also been shown in studies to be beneficial in adolescent and pediatrics patients with various types of tumors, including soft tissue sarcoma, malignant germ cell tumors, and chondrosarcomas.

The impact of HT in DDR is another alternative to improve the current radiotherapy strategy, and it can significantly radiosensitize the tumor cells. According to earlier studies, HT stimulates the ATM and γ-H2AX pathways and increases the expression of p53 (158, 159). As a result, HT induction is an important pathway in DDR and plays an early role in DDR responses. Another intriguing study found that HT has a direct beneficial effect in combination with DDR-targeted therapy by inhibiting the homologous recombinant repair pathway. In addition, it also deactivates the NHEJ pathway by suppressing the interaction between Ku80 and BRCA2 at DSB damage sites (160). A clinical trial is underway with HT and Olaparib combination therapy for BC patients (NCT03955640). The hypothesis is that HT could modify the immune system *via* systemic treatments, promoting the expansion of the MHC class I. Published reports demonstrated that HT induces the significant infiltration of cytotoxic T, B, and NK cells (161, 162, 245). The preceding studies demonstrated that combining HT with immuno- and radiation therapy may improve treatment efficacy. Mild HT is one of the potential adjuvant treatments to overcome tumor hypoxia. To gain a more precise understanding, we must investigate the molecular-level relationship between HT and tumor oxygenation in depth. Furthermore, to identify therapeutic targets and understand the underlying mechanisms (HIF pathways, ROS, heat shock proteins, and EMT) of heat resistance pathways that could be used as therapeutic targets in cancer patients (218–220).

### 3.5 Anti-angiogenic therapy

As already discussed in Section 2.1, the role of angiogenesis in cancer survival and progression, we can estimate that targeting the angiogenesis could be a possible approach to combat TNBC. While reviewing the literature, we found that several angiogenesis inhibitors are clinically available against different types of advanced solid cancers. These inhibitors are generally either monoclonal antibodies or small molecule-based tyrosine kinase inhibitor, which target the VEGF and receptors. Angiogenesis inhibitors act by blocking the activity and expression of pro-angiogenic factors, secreted by tumor cells by targeting their receptors. Consequently, these inhibitors reduce the amount of nutrients available for tumor growth, and promote tumor vasculature normalization, and increase the delivery of cytotoxic chemotherapy (246–248). Unfortunately, these angiogenesis inhibitors failed to respond against BC when comparing the patient’s survival outcome to that of other solid tumors. Although research is ongoing, several clinical trials are underway to explore the angiogenesis inhibitors clinical outcomes in BC and TNBC patients.

In clinical trials, in the subgroup analysis, TNBC patients had shown a significant improvement in overall response rate in the E2100 and Avado trials; however, no statistical differences were observed in the Ribbon 1 trial, in which bevacizumab, humanized anti-VEGF monoclonal antibody, was given in combination with either second-line treatment by using chemotherapy or bevacizumab plus paclitaxel for first-line treatment or bevacizumab is added to neoadjuvant chemotherapy (249–251). In these three trials, 684 patients with TNBC were enrolled, and a meta-analysis was performed. In these three trials, 684 patients with TNBC were enrolled, and a meta-analysis was performed. This exciting study has clearly shown a marginal increase in progression-free survival. The overall objective response rate was also statistically increased, and there was a trend towards improved overall survival (226). Similarly, several other monoclonal antibodies, such as ramucirumab, have also been studied in several clinical trials, but no improvement in the overall survival of patients has been observed (227).

Besides, few small molecule-based tyrosine kinase inhibitors were studied in several clinical trials. There are several inhibitors such as bevacizumab, lenvatinib, apatinib, cabozantinib have been shown a positive clinical response in BC patients including TNBCs which are summarized in **Table 1**. In addition, a few other examples include, sorafenib, vandetanib, sunitinib, axitinib, pazopanib and cediranib, which are approved in several other cancers, like advanced renal cell carcinoma, hepatocellular carcinoma, soft-tissue sarcoma, gastrointestinal stromal tumors, advanced pancreatic neuroendocrine tumors, medullary thyroid carcinoma etc. Till now, these inhibitors were studied either as alone or in combination of first- and second-line treatment in various studies, but no significant improvement in overall survival in BC patients has been observed. All these inhibitors generally target the classical angiogenic pathway by targeting VEGF and VEGFR, and gave suboptimal results (228, 229). Thus, in our view, there is a need to explore novel anti-angiogenic approaches, such as targeting pericytes for vascular normalization, miRNA utilization and usage of immunotherapeutic drugs.

### 3.6 Photodynamic therapy

Another emerging and constantly developing method to treat cancer is photodynamic therapy (PDT), which involves using low to medium-energy monochromatic light to photo-excite subsequently applied photosensitizers (PS) interacting with the oxygen and producing ROS. The interaction between light and tissue is *via* absorption, scattering, reflection and refraction. Tissue’s optical properties determine the distribution of treatment light, as most of the light is transmitted at near-infrared wavelengths. PDT uses light with a wavelength of 600-800 nm, and it is a well-known fact that light with longer wavelengths has been absorbed to a greater extent; therefore, one of the limitations of PDT is its therapeutic depth, which is less than a centimeter (252–254).

At its early stage, PDT is well established and accepted in dermatology such as non-melanoma skin cancers, pre-malignant conditions like actinic keratosis and Bowen’s disease (255). Besides, it is also accepted in non-dermatologic condition like head and neck cancer (256), low grade prostate cancer (257) and pancreatic cancer (258, 259). But now, there have been reports describing PDT as suitable options for treating cutaneous metastases from BC as well as primary BC (239, 260). PDT combined with traditional antitumor therapies show much promising effect in improving patient outcome and reducing the unwanted side effects. The combination of light with rhodamine 123 and its platinum complex, indocyanine green (261), meso-tetra hydroxyphenyl chlorine and zinc phthalocyanine has been proven very effective in *in -vitro* studies (262–265). Recently, Chou et al. study the effect of combination of PDT and bio reductive therapy in targeting TNBC with an aptamer functionalized nano formulation (23). This new therapeutic strategy, which utilized the combination of protoporphyrin IX and tirapazamine, performed well in both hypoxia and normoxia, and hence could be a promising medical procedure for effective treatment of TNBC (**Table 1**). In summary, the synergistic effect of PDT and traditional therapies could enhance the therapeutic effect and even can prove to be a better way to tackle TNBCs.

### 3.7 Adoptive cell therapy and cancer vaccines

Recently, adoptive cell therapy (ACT) and cancer vaccines have been proposed as future therapy approaches, which can cure various cancer stages, including TNBC. Adoptive cell therapy in TNBC mainly covers three types of ACT which include three types of therapy: tumor-infiltrating lymphocyte (TILs), engineered T cell receptor, and chimeric antigen receptor therapy (CAR-T) which is strongly correlated with the infiltration of T-cells in TNBC (266). These all ACT based on similar principles where patients’ natural t-cells have been modified genetically in ex-vivo condition and injected back into the patient’s body to make them tumor antigen-specific and accelerate their ability to kill cancer cells by triggering the cytotoxic immune response (266, 267). CART-T cells improve the effective tumor transport of engineered activated T-cells and overcome antigenic heterogeneity and the broad repertoire of immune escape mechanisms occurring in advanced TNBC. However, certain issues need to be addressed, such as identifying tumor-specific antigens (TSAs) rather than tumor-associated antigens (TAs) and optimizing the adverse effects of cell lysis for immune hyper-activation (215). Currently, CAR-T cell therapies have been FDA-approved for the treatment of various cancer-type patients, including TNBC, and a considerable number of clinical trials are testing CAR constructs against multiple tumor antigens in TNBC, which are summarized in **Table 1**.

Cancer vaccines also target TAs to accelerate tumor-specific immune responses through active immunization by generating cytotoxic CD8+ T-cell (CTLs) and other effector immune responses such as NK and dendritic cell responses (266). These vaccines consist of either peptides, carbohydrates, recombinant DNA or RNA, whole cells, or dendritic cells (DC), which summarizes in **Table 1**. In addition, neoantigen vaccines use peptides that are specific to mutations in the tumor and not present in normal cells, therefore have been shown to elicit robust immunogenic responses because of high tumor mutational burden (TMB) and further activates tumor antigen specific CD8+ and CD4+ T cells (266). Emerging evidence suggests that these cancer vaccines, in combination with ICI and chemotherapeutic agents, may boost the anti-tumor immune response. The current clinical trials using cancer vaccines in combination with ICI and chemotherapeutic agents are summarized in **Table 1**.

### 3.8 Antibody drug conjugates

ADC are immunoconjugative drugs which are specifically engineered by using three pre-defined immune components a) cytotoxic drugs, b) a chemical linker moiety and c) a humanized monoclonal antibody specifically recognizing neoplastic epitopes on tumor cells and overexpressed definite antigens {trophoblast cell surface antigen 2 (trop-2), receptor tyrosine kinase-like orphan receptor (180), human epidermal growth factor receptor (HER) etc.} (215, 268). These ADC drugs are degraded once it recognizes and conjugates with specific antigens in the highly acidic metabolic TME (268). ADC’s high target specificity and potency feature defines its novelty in personalized therapeutic approaches. Emerging evidence also suggests that a high therapeutic index compared to traditional chemotherapies and their specificity against selective tumor populations make the ADCs a promising partner for targeted agents in combination therapies (268, 269). However, several preclinical and clinical data have shown and suggested high pharmacological properties and improved survival benefits, several limitations still need to be improved, such as recognition of specific binding antigens, optimization of the drug-to-antibody ratio (DAR) and release of the chemical linker in tumor cells and their toxicities etc. Song Hua et al., 2010 have shown that novel anti-HIF-1α ADC nano micelles filled with paclitaxel precisely target and selectively kill the stomach cancer cells having high expression of HIF-1α and suggesting that HIF-1 ADC could be great potential in various clinical settings (270). Several clinical studies have so far been ongoing based on preclinical antitumor activity in both neoadjuvant and metastatic settings in the TNBC cohort, and trop-2 targeted sacituzumab govitecan is the first FDA-approved ADC for the mTNBC treatment (215, 269). **Table 2**. summarizes the ongoing clinical trials of ADCs and their analogues in locally advanced or metastatic TNBC.

**Table 2:** Development of antibody-drug conjugates (ADC) and ongoing clinical trials for TNBC treatment.

S.No.	Treatment	Target	Cleavable linker	TNBC cases	Cohort	Clinical Phase	Status	References
1.	Sacituzumab govitecan	Trop-2	SN-38	108	mTNBC	II	ORR: 33.3%; 5.5 mo.	(211, 215, 269)
2.	Datopotamab deruxtecan	Trop-2	Deruxtecan	44	mTNBC	I	Recruiting	(215)
3.	SKB264	Trop-2	Moderate cytotoxic belotecan-derivative	48	mTNBC	I-II	ORR: 35.3%	(211, 215)
4.	Mirvetuximab soravtansine	Folate receptor *α*	Tubulin-disrupting maytansinoid DM4	44	TNBC	I	Active	(215)
5.	Ladiratuzumab vedotin (SGN-LIV1a)	Zinc transporter LIV-1	Monomethyl auristatin E (MMAE)	310	mTNBC	1b/II	Recruiting	(211, 215, 269)
6.	NBE-002	ROR1	Anthracycline-derivative PNU-159682	100	TNBC	I/II	Recruiting	(215)
7.	VLS-101	ROR1	Monomethyl auristatin E (MMAE)	210	TNBC	II	Recruiting	(215)
8.	CAB-ROR2-ADC (BA3021)	ROR2	Conditionally active biologic (CAB)	120	TNBC	I/II	Recruiting	(215, 269)
9.	Anti-CA6-DM4 immunoconjugate (SAR566658)	CA6	DS6	23	mTNBC	II	completed	(269)
10.	Camidanlumab tesirine	CD25	Pyrrolobenzodiazepine	44	mTNBC	I	Recruiting	(215)
11.	Praluzatamabravtansine	Cd166	Tubulin-disrupting maytansinoid DM4	125	mTNBC	II	Recruiting	(215)
12.	Vobramitamab duocarmazine (MGC018)	CD276 (B7-H3)	Duocarmycin	143	mTNBC	I/II	Recruiting	(215)
13.	Anti-EGFR-immunoliposomes-DOX	EGFR	Doxorubicin	48	TNBC	I	ORR: 33%; PFS: 12mo.	(211, 215)
14.	AVID 100	EGFR	Cleavable linker with DM1	90	TNBC	Ia/Iib	Terminated	(215, 269)
15.	Trastuzumab dreuxtecan	HER2	Topoisomerase I inhibitor	278	mTNBC	II	Recruiting	(215, 269)
16.	Patritumab dreuxtecan	HER3	Topoisomerase 1 inhibitor payload, an exatecan derivative (DXd)	120	mTNBC	I	Recruiting	(215)
17.	Anetumab Ravtansine	Mesothelin (MSLN)	Maytansinoid tubulin inhibitor DM4	173	TNBC	Ib	Active/not recruiting	(215)
18.	Cofetuzumab peledotin	Protein tyrosine kinase 7	Auristatin	18	mTNBC	I	ORR: 16.7%; mPFS: 2mo.	(215)
19.	Enfortumab vedotin	Nectin-4	Monomethyl auristatin E (MMAE)	288	mTNBC	II	Recruiting	(211, 215)
20.	BT8009	Nectin-4	Monomethyl auristatin E (MMAE)	329	TNBC	I/II	Recruiting	(215)
21.	TH1902 peptide	Sortilin	Docetaxel-peptide conjugate	70	mTNBC	I	Recruiting	(215)
22.	Rovalpituzumab Tesirine	Delta like protein 3 (DLL-3)	Cytotoxic pyrrolobenzodiazepine (PBD)	182	TNBC	I	Active/not recruiting	(211)

mTNBC, metastatic TNBC; pCR, Pathological complete response; PFS, Progression free survival; mPFS, mean progression free survival; ORR, Objective response rate; mOS, Mean objective survival, ITT, Intention to treats; mo., month.

### 3.9 Combination therapies

TNBC lacks expression of some generalized targeted receptors such as estrogen, progesterone, and HER2 receptors, making it difficult to target with conventional therapies. However, combination therapy involves the simultaneous use of multiple treatment modalities, such as chemotherapy, targeted radiotherapy, and immunotherapies, to enhance efficacy and overcome resistance mechanisms by targeting multiple signaling pathways and tumor vulnerabilities. The combination treatment approaches mostly involved tailored strategies based on individual patient characteristics and the tumor’s molecular profile, leading to precise therapy to improve patient outcomes. Paclitaxel and nab-paclitaxel are among the frequently used chemotherapy options, but their resistance is one of the major reasons for the failure and relapse of TNBC (271). Therefore, currently, several clinical trials are undergoing where the combination of paclitaxel or nab-paclitaxel with immune checkpoint inhibitors such as atezolizumab, cobimetinib, or PARP, AKT, PI3K, or VEGF inhibitors has been administered, leading to a significant increase in mean objective survival and response rate. Besides, the combination of chemotherapy and immune checkpoint inhibitors followed by adjuvant therapy are also under clinical trials, exhibiting significant positive responses. Similarly, the combination of anti-angiogenic therapy like lenvatinib, apatinib etc., with several inhibitors also exhibits positive responses in undergoing clinical trials. Ongoing research and clinical trials continue to explore innovative combination regimens, offering hope for improved survival rates and a brighter future for TNBC patients, and we have summarized such clinical trials revolving around combination therapy in **Table 3**.

**Table 3:** Current combination treatments in TNBCs.

S. No.	Treatment	TNBC cases	Cohort	Clinical Phase	Status	References
A.	Current clinical trials of ICIs involving patients with metastatic/early stage TNBC					
1.	Nab-paclitaxel+atezolizumab	33	mTNBC	Ib/III	ORR: 39.4%, mPFs: 5.5mo, mOS 14.7mo.	(203, 272)
2.	Ipatasertib and atezolizumab plus either nab-paclitaxel	26	mTNBC	Ib	ORR: 73%	(203)
3.	Ladiratuzumab vedotin + pembrolizumab	26	mTNBC	Ib/II	ORR: 54%	(203)
4.	Durvalumab + trastuzumab deruxtecan	21	mTNBC	Ib/II	ORR: 66.7%	(203, 273)
5.	Eribulin + pembrolizumab	167	mTNBC	Ib/II	ORR: 23.4, mPFS: 4.1mo, mOS: 16.1mo.	(203, 274)
6.	Atezolizumab+taxanes+MEKi	902	Locally advanced/mTNBC	II	Active/ORR: 29%-34%	(275)
7.	Pembrolizumab +MEKi	12	Locally advanced/mTNBC	I/II	Recruiting	(275)
8.	Cobimetinib and atezolizumab + either nab-paclitaxel/paclitaxel	63	mTNBC	II	ORR: 31.7%	(203, 276)
9.	Entinostat + atezolizumab	40	mTNBC	II	ORR: 10%; mPFS: 1.68mo; mOS: 9.4mo.	(203)
10.	Lenvatinib + pembrolizumab	31	mTNBC	II	ORR: 29%	(203)
11.	Paclitaxel + atezolizumab/placebo	651	mTNBC	III	ORR in ITT: 53.6 *vs*. 47.5%	(203, 277)
12.	GX-17 + pembrolizumab	30	mTNBC	Ib/II	ORR: 13.3%	(203)
13.	Nab-paclitaxel+atezolizumab/placebo	902	mTNBC	III	ORR: 45.9%	(277)
14.	Pembrolizumab + nab-paclitaxel/paclitaxel/gemcitabine/carboplatin	882	mTNBC	III	PFS: 9mo.	(19, 275)
15.	Pembrolizumab + gemcitabine/carboplatin	87	mTNBC	II	Pending	(275)
16.	Pembrolizumab + eribulin mesylate	167	mTNBC	Ib/II	ORR: 25%; PFS: 4.1mo.	(275)
17.	Nivolumab after Cyclophosphamide/cisplatin/doxorubicin	66	mTNBC	II	ORR: 35% (Doxorubicin);	(275)
18.	Atezolizumab +nab-paclitaxel	900	Locally advanced/mTNBC	III	ORR: 53%; OS: 25mo	(166, 275)
19.	Atezolizumab + paclitaxel	600	Locally advanced/mTNBC	III	Pending	(275)
20.	Atezolizumab + gemcitabine/carboplatin or capecitabine	540	Locally advanced/mTNBC	I	Recruiting	(275)
21.	Atezolizumab + paclitaxel followed by atezolizumab +AC or EC	2,300	Locally advanced	III	Recruiting	(275)
22.	Neoadjuvant pembrolizumab + paclitaxel and AC	114	Locally advanced	II	Recruiting	(275, 278)
23.	Neoadjuvant pembrolizumab + chemotherapy combination (Nab-paclitaxel, paclitaxel, doxorubicin, Cyclophosphamide, carboplatin	60	Locally advanced	I	Completed, pCR: 60%	(275)
24.	Neoadjuvant pembrolizumab + paclitaxel-carboplatin followed by adjuvant pembrolizumab	1,174	Locally advanced	III	pCR: 64.8%	(279)
25.	Paclitaxel ± Pembrolizumab followed by adjuvant thaerpy	114	Early Stage	II	pCR: 60% *vs.* 22%	(203)
26.	Nab-paclitaxel+durvalumab/placebo followed by endocrine therapy+durvalumab/placebo	174	Early Stage	II	pCR in ITT: 53.4% *vs*. 44.2%	(203)
27.	Pembrolizumab+anthracycline+taxane-based chemotherapy ± carboplatin followed by adjuvant chemotherapy	60	Early Stage	ib	pCR overall: 60%	(203)
28.	Nab-paclitaxel+atezolizumab/placebo followed by adjuvant chemotherapy+atezolizumab/placebo	313	Early Stage	III	pCR in ITT: 58% *vs*. 41%	(203, 280)
29.	Anthracycline, taxane and carboplatin+Pembrolizumab/placebo followed by adjuvant chemotherapy/endocrine therapy	1,174	Early Stage	III	pCR: 63% *vs.* 55%	(203, 281)
30.	Nab-paclitaxel+acarboplatin ± atezolizumab	280	Early Stage	III	pCR in ITT: 43.5% *vs*. 40.8%	(203)
31.	Neoadjuvant atezolizumab+paclitaxel+carboplatin followed by atezolizumab +AC or EC	1520	Early Stage	III	Recruiting	(203)
32.	Atezolizumab + carboplatin + nab-paclitaxel	278	Early/Locally advanced/mTNBC	III	Active/not recruiting	(215)
33.	Atezolizumab + neoadjuvant chemotherapy	1550	TNBC	III	Active/not recruiting	(215)
34.	Atezolizumab + nabpaclitaxel	184	Locally advanced/mTNBC	III	Active/not recruiting	(215)
35.	Atezolizumab + chemotherapy	572	Locally advanced/mTNBC	III	Recruiting	(215)
36.	Atezolizumab + adjuvant anthracycline/taxane based therapy	2300	Locally advanced/mTNBC	III	Recruiting	(215)
37.	Atezolizumab + ipataseritib and paclitaxel	242	mTNBC	III	Active/not recruiting	(215)
38.	Avelumab as adjuvant or post-neoadjuvant	474	Locally advanced/mTNBC	III	Active/not recruiting	(215)
39.	Camrelizumab + Chemotherapy	581	Locally advanced/mTNBC	III	Recruiting	(215)
40.	Serplulimab + chemotherapy	522	Locally advanced/mTNBC	III	Not recruiting	(215)
41.	Toripalimab + nab-paclitaxel	531	Locally advanced/mTNBC	III	Recruiting	(215)
42.	Carelizumab + nab-paclitaxel + apatinib *vs*. Carelizumab+nab-paclitaxel *vs.* nab-paclitaxel	80	Locally advanced/mTNBC	III	Recruiting	(215)
43.	TQB2450 + anlotinib hydrochloride/paclitaxel	332	TNBC	III	Not recruiting	(215)
44.	Anti-Globo-H-Vaccine adagloxad simolenin (OBI-822)/OBI-821	668	Early Globo-H+ TNBC	III	Recruiting	(215)
B.	Current trials of combination chemotherapeutic agents involving patients with metastatic/early stage TNBC					
1.	Ixabepilone+capecitabine *vs*. capecitabine	443	mTNBC	III	PFS: 4.2 *vs*. 1.7mo.; OS: 9.0 *vs*. 10.4 mo.	(215)
2.	Pacitaxel+carboplatin *vs*. cyclophosphamide+epirubicin+fluorouracil+docetaxel	647	TNBC	III	DFS: 86.5 *vs*. 80.3%	(215)
3.	Docetaxel+epirubicin ± lobaplatin	125	TNBC	II	pCR: 93% *vs*.73%	(215)
4.	Cisplatin+gemcitabine *vs*. paclitaxel+gemcitabine	236	mTNBC	III	PFS: 7.7 *vs*. 6.47mo.	(215)
C.	Current trials of PARP inhibitor involving patients with metastatic/early stage TNBC					
1.	Veliparib/Paclitaxel/carboplatin *vs*. Paclitaxel/carboplatin *vs*. Paclitaxel	634	Early Stage	III	pCR: 53% *vs*.58%	(215)
2.	Veliparib+carboplatin	72	Locally advanced/mTNBC	II	pCR51%*vs*.26%	(215)
3.	Paclitaxel/carboplatin ± olaparib	527	Early stage TNBC	II/III	pCR15 to 20%	(275)
4.	Olaparib + Pembrolizumab *vs*. carboplatin/gemcitabine	932	Locally advanced/mTNBC	II/III	Recruiting	(282)
5.	Olaparib + durvalumab	17	mTNBC	I/II	ORR: 58.8%; mPFS: 4.9mo; mOS: 20.5mo.	(203, 283)
6.	Niraparib + pembrolizumab	45	mTNBC	I/II	ORR: 29.0%; mPFS 2.3mo.	(203, 284)
7.	Atezolizumab + olaparib	81	Locally advanced/mTNBC	II	Active/Not-recruiting	(275)
8.	Iniparib+ gemcitabine/carboplatin	80	Early Stage TNBC	II	pCR: 36%	(215)
D.	Current trials of AKT inhibitor involving patients with metastatic/early stage TNBC					
1.	Paclitaxel ± ipatasertib	450	Locally advanced/mTNBC	III	PFS: 9.3mo; ORR: 47%	(282)
2.	Paclitaxel+ipatasertib or placebo	124	Locally advanced/mTNBC	II	PFS: 6.2 *vs*. 4.9 mo.	(215)
3.	Ipatasertib+paclitaxel or placebo	151	Early stage TNBC	II	pCR: 17% *vs*. 13%	(215)
4.	Paclitaxel ± capivasertib	800	Locally advanced/mTNBC	III	Recruiting	(282)
5.	Paclitaxel + capivasertib or placebo	140	mTNBC	II	PFS: 5.9 *vs*. 4.2 mo.	(215)
6.	Paclitaxel/ipatasertib/Atezolizumab *vs*. Paclitaxel/ipatasertib *vs*. Paclitaxel	450	Locally advanced/mTNBC	III	Recruiting	(282)
E.	Current trials of PI3K inhibitor involving patients with metastatic/early stage TNBC					
1.	Nab-paclitaxel ± alpelisib	566	Locally advanced	III	Pending	(282)
2.	Paclitaxel+buparlisib or placebo	416	mTNBC	II/III	PFS: 8.0 *vs*. 9.2 mo.	(215)
3.	Camrelizumab+apatinib or intermittent apatinib	40	mTNBC		PFS: 3.7 *vs*. 1.9 mo.	(215)
F.	Current trials of VEGF/VEGFR inhibitor involving patients with metastatic/early stage TNBC					
1.	Paclitaxel+carboplatin *vs*. cyclophosphamide+carboplatin+/or bevacizumab	443	mTNBC	III	pCR: 93.5% *vs*. 73.0%	(215)
2.	Anthracycline+taxane± bevacizumab	493	TNBC	III	pCR: 50%	(215)
3.	Paclitaxel+doxorubicin+ bevacizumab±;carboplatin	315	TNBC	II	pCR: 53% *vs*. 36.9%	(215)
G.	Current trials in combination with cancer vaccine involving patients with metastatic/early stage TNBC					
1.	Pembrolizumab+PX-410	20	TNBC	I	Active/ Not-recruiting	(215)
2.	Pembrolizumab+p53-MVA	11	TNBC	I	Active/ Not-recruiting	(215)
3.	Durvalumab+PX-410	22	TNBC	I	Active/ Not-recruiting	(215)
4.	Durvalumab+neo-antigen DNA vaccine	18	TNBC	I	Active/ Not-recruiting	(215)
5.	Durvalumab+nab-paclitaxel+neo-antigen DNA vaccine	70	TNBC	II	unknown	(215)
6.	Atezolizumab+neo-antigen DNA vaccine	272	TNBC	I	Active/ Not-recruiting	(215)

ICIs, Immune checkpoint inhibitors; mTNBC, metastatic TNBC; pCR, Pathological complete response; PFS, Progression free survival; mPFS, mean progression free survival; ORR, Objective response rate; mOS, Mean objective survival, ITT, Intention to treats; mo., month.

## 4 Hypoxia: a main culprit to nullify the various cancer treatment strategies

Normal tissues generally require a steady supply of oxygen and nutrients to stay alive and remove waste through metabolism. The solid tumor, unlike normal tissue, has dysfunctional vasculature (285, 286). The rate of tumor progression, stroma composition, and pathological vasculature all contribute to a hypoxic environment in the tumor microenvironment, which impairs immune cell function. Furthermore, hypoxia creates selection pressure by promoting cell growth alongside genetic machinery having malignant potential (287). As a result, hypoxia causes EMT, which leads to cell mobility and metastasis (288, 289). Moreover, metabolism of tumor cell reforms after hypoxia, leading to cell quiescence (30, 64). This condition alters transport or distribution and is resistant to radiotherapy, chemotherapy, immunotherapy, and adjuvant therapy (31, 290). Chemotherapy and radiotherapy affect proliferating tumor cells, especially in normoxic states, but hypoxic cells survive these antineoplastic therapies. Thomlinson and Gray et al. previously proposed that hypoxia is a “diffusion-limited chronic hypoxia” (291).

The top preclinical evaluation studies demonstrated increasing tumor oxygenation by modifying oxygen delivery by allowing tumor-bearing rodent models to inhale either (95% O_2 + _5% CO_2_) carbogen or 100% oxygen. According to the data, the tumor grew significantly after radiation (292). Many preclinical studies previously reported that cancer cells were more malignant in hypoxic conditions. Earlier, Young et al. *in-vitro* studies demonstrated that cells were kept for 18–24h in hypoxic conditions and injected into the mice (293). In such cases, injected cells reach the lungs and form lung nodules; additionally, they reported that the level of hypoxia in the primary tumor directly increases the number of metastases in tumor-injected mice, regardless of whether the hypoxia was natural or induced (294, 295). According to the previous report, two separate clinical trial studies on how hypoxia influences the malignant progression of cancer cells were conducted. The study’s findings revealed that the oxygenation status of the patients was assessed using the Eppendorf electrode before the regimen. Previously, only one study on a cervix cancer patient who underwent surgery was reported (296). In the other group, most soft-tissue sarcoma patients underwent surgery (297). In such cases, injected cells reach the lungs and develop lung nodules. In addition, they also reported that the level of hypoxia in the primary tumor directly aggravated the number of metastases in tumor-injected mice regardless of whether that hypoxia was natural or induced (294, 295). Based on the previous report, two separate clinical trial studies were conducted on how hypoxia influences cancer cells’ malignant progression. The study outcome revealed that the oxygenation status of the patient’s assessed by applying the Eppendorf electrode before the regimen. Earlier, only one study was reported on a cervix cancer patient who underwent surgery (296). In the other group, most patients with soft-tissue sarcoma underwent surgery (297). Both studies found that patients who had previously received oxygenation treatment had an overall higher survival rate. Patients with higher levels of hypoxia had significantly worse survival outcomes based on their pretreatment oxygenation status (296, 297). Later clinical studies revealed that using an eppendorf electrode causes significant hypoxia in leiomyomas, myometrium, and leiomyosarcomas, all originating in premenopausal women (298). Solid tumors directly contribute to cancer’s malignant properties and are a hallmark of hypoxia (299, 300). It is well understood that HIFs instantly activate tumors through HIF transcription factors, which promote changes in the expression of VEGF and CAIX levels, both of which are required for unstable and anaerobic energy production (301). In general, HIFs promote the expression of multiple genes involved in metabolic management, pH balance, angiogenesis, and cell apoptosis, all of which contribute to tumor survival.

**Figure 5:**
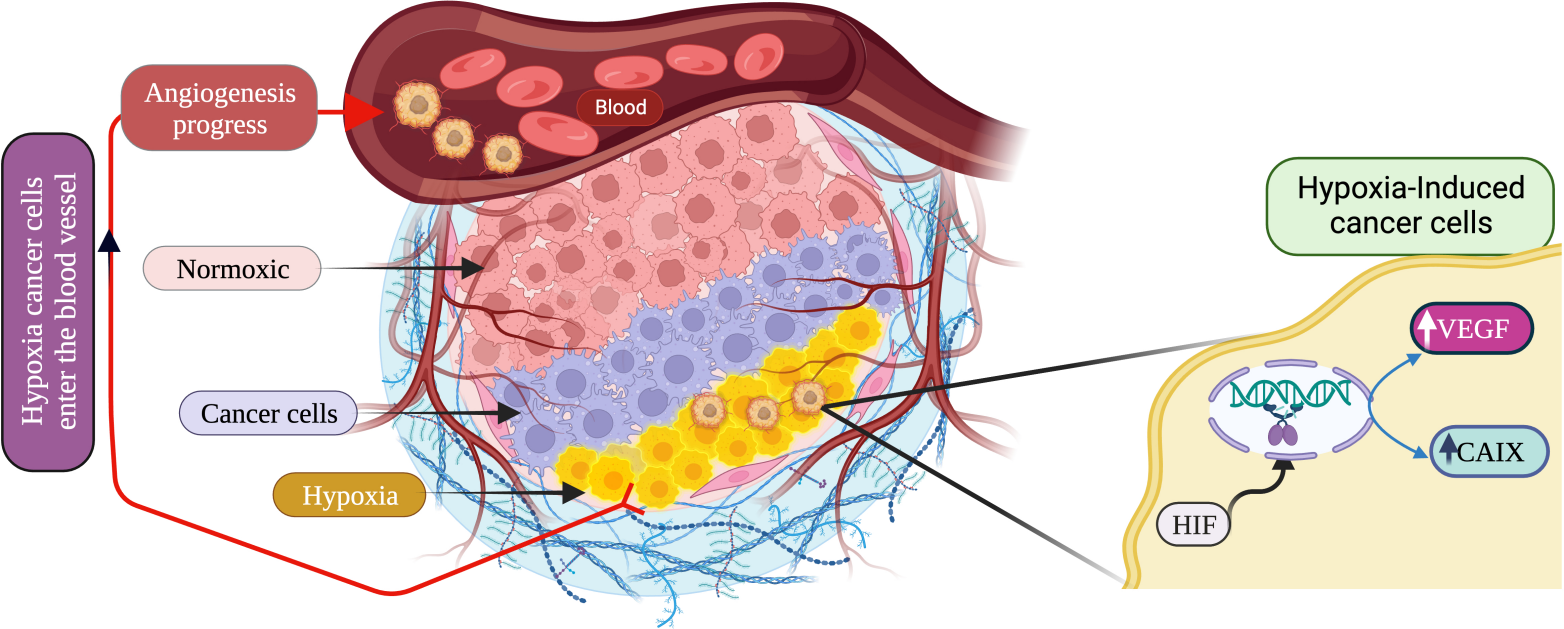
Schematic description of hypoxic cancer microenvironment and angiogenesis progression. (Created *via* BioRender.com).

As a result, **Figure 5** clearly explains the mechanism, as mentioned earlier. HIFs play a role in tumor blood recovery *via* vascular protection and promote nutrient supply to solid tumors, which become one of the most difficult to treat, leading to resistance to chemotherapy, radiotherapy, immunotherapy, and adjuvant therapy.

### 4.1 Targeting hypoxia: a new tactic to improve current TNBC therapy

A key goal of TNBC therapy has been to target hypoxia, which inhibits several tumor characteristics, including metastasis, radio-resistance, and chemoresistance (302, 303). A study that has been published suggests that many hypoxia-related genes (HRGs) and their mediators, HIFs, may be used as therapeutic targets and prognostic indicators in BC (34, 303).

HIF1α is a well-established key target regulating the TNBC, and its expression is regulated by various signaling pathways like NF-κB, PI3K/Akt/mTOR, RAS-RAF-ME-ERK and JAK-STAT. Studies have found that under hypoxic conditions, HIF1α induced STAT3 *via* JAK or adenylate receptor 2B pathway, which upregulates the IL-6 and NANOG to maintain the CSC phenotype and also enhances the production of VEGF, required for the self-renewal ability of CSCs (79). Studies have also demonstrated that in hypoxic conditions, HIF-1α activates the Sonic Hedgehog signaling pathway to induce the production of CSC markers in cholangiocarcinoma cells which can be blocked by HIF-1α inhibition (304). Emerging studies also demonstrated that HIF1α suppress the ERK activity and induces the P38 activity, which further upregulates NANOG and KLF4 to promote the development of breast CSCs. Several studies have been conducted to identify targetable molecules from these signaling pathways that characterize various inhibitors or drug molecules (304, 305). The astonishing fact is that some of these signaling pathways can be targeted by already approved therapeutics or inhibitors under clinical trials alpelisib is an approved inhibitor while buparlisib is under clinical trial, and both inhibit class I PI3K. Similarly, several inhibitors target VEGF, EGFR, PARP, and cell cycle and have shown significant outcomes in TNBC patients. **Table 4** summarizes potential inhibitors and drug molecules against molecular targets and signaling pathways involved in the progression of hypoxia-induced TNBC.

**Table 4:** Potential inhibitors and drugs for hypoxia induced TNBC.

S. No.	Inhibitors/Drugs	Mechanism of Action	References
A.	Tropomycin receptor kinase (TRK) Inhibitors		(203, 215)
1.	Larotrectinib	Binds to Trk and prevent neurotrophin-Trk interaction and Trk activation	
2.	Selitrectinib	Inhibitor of Trk receptors	
3.	Repotrectinib	Inhibitor of Trk receptors	
B.	Human epidermal growth factor receptor (HER) Inhibitors		(203, 215)
1.	Netatinib	Inhibit Growth factor receptors	
C.	Phosphoinositide 3-kinase (PI3K) Inhibitors		(203, 215)
1.	Alpelisib	Inhibit class I PI3K p110α	
2.	Taselisib	PI3K Inhibitor targeting PI3Kα/δ/γ	
3.	Buparlisib	Inhibits class I PIK3 in ATP-competitive manner	
4.	Sapanisertib	Inhibitor of raptor-mTOR (TOR complex 1 or TORC1) and rictor-mTOR (TOR complex 2 or TORC2)	
5.	Ipatasertib	Inhibit PI3K pathway	
6.	Uprosertib	Binds to and inhibits the activity of Akt, which may result in inhibition of the PI3K/Akt signaling pathway	
7.	Samotolisib	Inhibitor of certain class I phosphoinositide 3-kinase (PI3K) isoforms and mammalian target of rapamycin kinase (mTOR) in the PI3K/mTOR signaling pathway	
8.	Copanlisib	Inhibit PI3K-α and PI3K-δ isoforms	
9.	Eganelisib	Inhibits gamma isoform of phosphoinositide-3 kinase	
10.	Gedatolisib	Inhibits both PI3K and mTOR kinases	
11.	GDC-0941	Inhibit PI3K pathway	
12.	NVP-BKM120 (BKM-120)	Inhibit PI3K pathway	
13.	BEZ235 (NVP-BEZ235)	Inhibit PI3K/mTOR pathway	
14.	GDC-0980	Inhibit PI3K/mTOR pathway	
D.	Protein kinase B (PKB/AKT) Inhibitors		(203, 215)
1.	Ipatasertib	Inhibits AKT pathway	
2.	Capivasertib	Inhibits AKT pathway	
E.	Mammalian target of rapamycin (mTOR) Inhibitors		(203, 215)
1.	Everolimus	Inhibits mTOR	
2.	Vistusertib	Inhibits mTOR 1/mTOR2	
3.	Gedatolisib	PI3K/mTOR inhibitor	
F.	Mitogen-activated protein kinase (MEK) Inhibitors		(203, 215)
1.	Trametinib	Inhibits MEK pathway	
2.	Binimetinib	Inhibits MEK pathway	
3.	Selumetinib	MEK 1/2 inhibitor	
G.	Cyclin-dependent kinase 4 and 6 (CDK4/6) Inhibitors		(22, 203, 215)
1.	Palbociclib	Inhibits CDK4/6	
2.	Abemaciclib	Inhibits CDK4/6; G0/G1 arrestor, induce chromatin condensation	
3.	Ribociclib	Inhibits CDK4/6; G0/G1 arrestor, induce apoptosis	
H.	Checkpoint kinase 1 (CHK1) Inhibitors		(22, 203, 215)
1.	LY2880070	Inhibits CHK 1	
2.	Prexasertib	Inhibits CHK 1 and induced Homologous recombination deficiency	
I.	WEE1 Inhibitors		(22, 203, 215)
1.	AZD1175	Inhibits WEE1	
2.	ZN-c3	Inhibits WEE2	
3.	MK1775	Inhibits WEE 1 kinase, G2/M arrestor; sensitize cells to cisplatin	
J.	Checkpoint kinase 2 (CHK2) Inhibitors		(203, 215)
1.	LY2606368	Inhibits CHK2	
K.	Androgen Receptors (AR) Inhibitors		(22, 306, 307)
1.	Bicalutamide	Inhibits AR	
2.	Enzalutamide	Inhibits AR	
3.	Abiraterone	Inhibits AR	
4.	Enobosarm	Inhibits AR	
5.	Darolutamide	Inhibits AR	
6.	17-DMAG	HSP-90 inhibitor, regulate the stability of AR	
7.	VT464	Involved in synthesis of AR	
L.	Atxia telangiectasia and Rad3-related (ATR) Inhibitors		(203, 215)
1.	Ceralasertib	Inhibits ATR	
M.	RAD51 Inhibitor		(203, 215)
1.	CYT-0851	Inhibits RAD51	
N.	Poly (ADP ribose) polymerase (127) Inhibitors		(22, 203, 215)
1.	Olaparib	Inhibit PARP	
2.	Talazoparib	Inhibit PARP	
3.	Veliparib	Inhibit PARP	
4.	Rucaparib	Inhibit PARP	
5.	Niraparib	Inhibit PARP	
6.	Pamiparib	Inhibit PARP	
7.	Fluzoparib	Inhibit PARP	
8.	Iniparib	Inhibit PARP1	
O.	Carbonic anhydrase IX (CAIX) Inhibitors		(308)
1.	SLC-0111	Inhibitor of carbonic anhydrases IX/XII; resulted in CSCs and EMT inhibition in TNBC cell lines	
2.	DTP348	CAIX inhibitor/radiosensitizer, inhibits HIF-1α in TNBC by targeting Hsp 90	
P.	Cell cycle Inhibitors		(203, 215)
1.	Trilaciclib	Inhibits CDK4/6; G)/G1 arrestor	
2.	Etoposide	Inhibits CDK4/6; G)/G1 arrestor	
3.	PF-06873600	Inhibitor of CDK4/6	
4.	Abemaciclib (Verginio)	Inhibitor of CDK4/7	
5.	Prexasertib	Inhibits CHK 1 and induced Homologous recombination deficiency	
Q.	Vascular endothelial growth factor/receptor (VEGF/VEGFR) Inhibitors		(203, 215)
1.	Anlotinib	Inhibitors of VEGFR 1/2 and FGFR 1/4	
2.	Apatinib	Inhibitors of VEGFR 2	
3.	Afatinib	Inhibitors of ErbB family of receptors (EGFR/ErbB1, HER2/ErbB2, ErbB3, and ErbB4)	
4.	Lenvatinib	Inhibitors of FGFR 3 and decreases the phosphorylation of downstream molecules of the FGF signaling pathway (such as FRS2, Erk, and p38 MAPK), and induced PARP cleavage	
5.	Erlotinib	Reduces VEGF promoter activity	
6.	Famitinib	inhibitor of targeting VEGFR2, PDGFR and c-kit	
7.	Pyrotinib	Irreversible pan-ErbB receptor tyrosine kinase inhibitor that targets hEGFR (HER) 1, HER2, and HER4	
8.	Bevacizumab	Inhibitors of VEGFR 2	
R.	Epidermal growth factor receptor (EGFR) Inhibitors		(22, 203, 215)
1.	Dasatinib	Block BCSCs enrichment and Src activation	
2.	Gefitinib	Inhibits AKT and MEK pathway	
3.	Sorafenib	Modulates the SHP-1/STAT3 axi	
4.	Nimotuzumab	Inhibitor of EGFR pathway and has low immunogenicity	
5.	Panitumumab	Inhibitor of EGFR pathway	
6.	SCT200	Inhibitor of EGFR pathway	
S.	g-Secretase Inhibitors		(215)
1.	AL101	Inhibitor of NOTCH 1, 2, 3, and 4	
2.	PF-03084014	Inhibitor of g-secretase inhibitor	
T.	AXL Kinase Inhibitors		(215)
1.	Bemcentinib	AXL kinase inhibitor; inhibits Axl phosphorylation	
U	Hedgehog pathway Inhibitors		(215)
1.	Vismodegib	Hedgehog (Hh) pathway inhibitor	
V.	CXCL8 and CXCR1/2 Inhibitors		(203, 215)
1.	Reparixin	Allosteric inhibitor of CXCR1, reduced the CSC content of human BC	
W.	Hypoxia-activated prodrugs (HAP) of DNA-damaging cytotoxins		
a.	DNA breakers		(309)
1.	Tirapazamine	Produces hydroxyl or and benzotriazinyl radicals as the DNA damaging reactive species in hypoxic cells	
2.	SN30000	Selective activation to a DNA-reactive radical species under hypoxia	
b.	DNA alkylators		(309)
1.	TH-302	Cellular reductases that generate a radical anion through 1-electron reduction	
2.	PR-104	Exploit hypoxia and HR defects in tumors, with translational implications for TNBC and other HR-deficient malignancies	
3.	SN30548	Exploit hypoxia and HR defects in tumors, with translational implications for TNBC and other HR-deficient malignancies	

**Figure 6:**
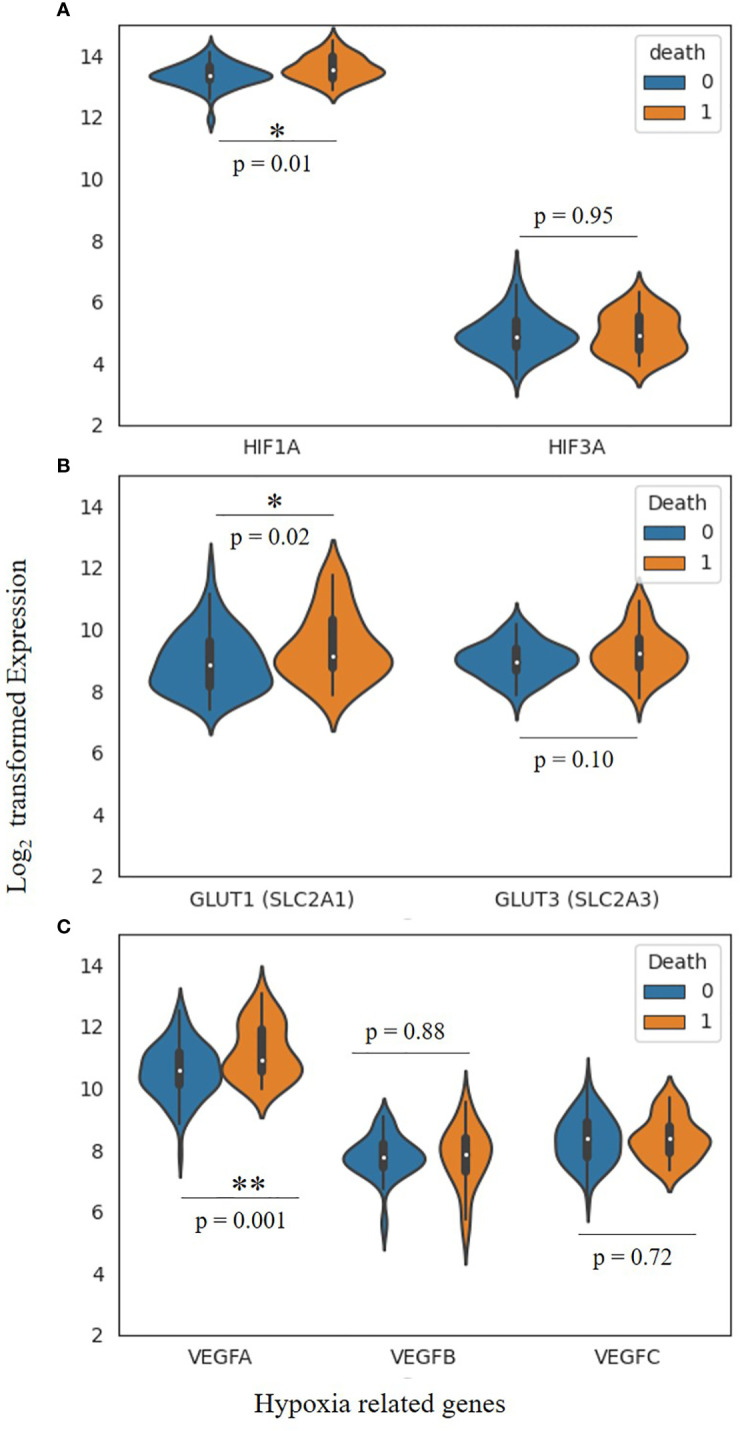
Hypoxia-related gene expression and morality in TNBC patients. **(A)** Violin plot exhibiting the expression of HIF-1α and HIF-3 α, **(B)** GLUT-1 and GLUT-3 Glucose transporters (genes SLC2A1 and SLC2A3) and **(C)** Vascular endothelial growth factors, VEGFA, VEGFB as well as VEGFC. Two-tailed T-test has been aplied for the significance (*, *P < 0.05*, **, *P < 0.005*). Cohort contains n=107 TNBC pateint samples (alive 79, dead 28).

TNBC patients exhibit higher mortality rates, and it has already been studied that overexpression of HIF-1α is associated with poor prognosis in various cancer (302). Here, we have explored a publicly available gene expression dataset (GSE103091, subseries GSE58812) to study the effect of hypoxia-related gene expression on mortality (303, 304). This dataset contains gene expression molecular subtyping of TNBC samples from 107 patients (78 alive and 29 dead). We have explored the expression of HIF and VEGFs and genes for glucose transporters. As shown in **Figure 6**, the overall presentation of HIF-1α is significantly higher in the patients who died due to TNBC than in the alive patients. However, there is no significant relationship between HIF-3α with the mortality.

Similarly, the expression of VEGF-A and GLUT-1 significantly (p= 0.001 and 0.02, respectively) differs in both cohorts. This study suggests a possible association between hypoxia-related gene expression and mortality. However, other factors like age, cancer grade, metastatic etc., haven’t been considered and may impact the conclusion. But hypothetically, there is a strong correlation between hypoxia-related gene expression and mortality, and it needs to be validated in larger cohorts.

### 4.2 The potential significance of targeting HIF-1α in different therapies

The association between HIF-1α and TNBC strongly suggests the possibility of novel targeted therapy in combination with chemotherapy, anti-angiogenic therapy, and immunotherapy for TNBC treatment (97, 102). HIF-1α reflects its potential to improve the current therapeutic outcome because of its extensive biological activities, particularly its function in angiogenesis, activation, and enhancement of tumor stem cells among other processes. The enrichment of BCSCs in tumors generated by various chemotherapeutic treatments is highly correlated with the increase of HIF-1, which is the major hurdle against chemotherapy. Clinical evidences support that some molecules like selenium, docosahexaenoic acid (DHA), eicosapentaenoic acid (EPA) in combination with low-dose chemotherapeutic agents significantly induced the degradation of HIF-1α and limits the BCSCs enrichment which may increase TNBC chemotherapy resistance (97, 310).

It is well documented that HIF-1 is stabilized in hypoxic conditions, and transcriptionally controls the lactate dehydrogenase A (LDHA) gene, which is associated to glycolysis and supports an acidic milieu. In patients with TNBC, this acidic milieu changes increases the CD8+ T cell counts and the generation of IFN, which is linked to a better clinical result and a stronger immunological response (311). Therefore, to improve the acidic microenvironment through HIF-1/LDHA targeting may restore the cytotoxic effect of CD8 cells to enhance the impact of immunotherapy in TNBC (97, 312). HIF-1α interaction with HDAC1 and concurrent PRC2 dependency epigenetically suppress the effector genes and induces the immune dysfunction in TNBC which results resistance to immunotherapy. A recent study in syngeneic and humanized TNBC mouse model has shown the efficacy of PD-1 blockade combined with HIF-1α and HDAC1 inhibition by PX478 and ENT respectively to reverse the anti-PD-1-resistant TNBC and significantly reduces tumor metabolic activity and metastasis (102, 313). Some clinical studies have showed that anti-angiogenic therapy alone is not recommended as the first-line treatment for metastatic TNBC since it increases the likelihood of TNBC invasion and metastasis. Consequently, inhibiting HIF-1 can enhance clinical efficacy by preventing invasion and metastasis-induced anti-angiogenic treatment. When used with the anti-angiogenic drug avastin, Guo et al. discovered that selenium with omega-3 polyunsaturated fatty acids decrease angiogenesis and metastasis *via* preventing COX-2 overexpression induced by HIF-1 (168). Furthermore, the anti-angiogenic drug bevacizumab hyperactivates the Wnt/β-catenin signaling in response to HIF-1α’s high expression in TNBC because of aberrant expression of frizzled 7 (Fzd7), a key receptor for Wnt/β-catenin signaling’s key receptor that induces cell invasiveness and metastasis (314, 315). Consequently, using an anti-Fzd7 antibody (SHH002-hu1) to target hypoxia adaptation-related proteins VEGFA and Glut1 expression as well as HIF-1 transcriptional activity will decrease TNBC cells’ acclimation to hypoxia and counteract the negative effects of anti-angiogenic medicines (314).

Under hypoxic conditions, irradiation can increase HIF-1 expression, which would lead to radio resistance (316). There are some confirmed reports suggesting that after radiation therapy, the availability of oxygen and glucose is increased in solid tumors, which activate HIF-1 and promote EMT that is HIF-1 dependent. Because of the translocating cells’ proximity to the blood arteries, which allows them to absorb nutrition and oxygen, tumor recurrence may be made easier (317, 318). The HIF-1 dependent translocation and migration of the surviving cells towards radio-protected blood vessels may indicate a specific role for HIF-1 in both local tumor recurrence and distant tumor metastasis after radiation therapy. Recent findings also suggest that HIF-1 inhibition utilizing HIF-1 inhibitors, may enhance radiosensitivity, chemosensitivity, immunosenstivity that could potentially provide advantages to methods of therapeutic treatment for hypoxic malignancies (316, 319). **Table 5** summarizes several potential synthetic compounds and natural products that have clinically proven to inhibit the HIF-1α activity at the transcriptional and translational level in TNBC, such as inhibiting the mRNA level of HIF-1α and their dimerization with HIF-1β as well as accelerating the degradation of the HIF-1α protein.

**Table 5:** Summary of related drugs/inhibitors targeting HIF-1α.

S.No.	Agents	Mechanism of action	Target gene/ Signaling	Therapeutic strategies	Pre-clinical/Clinical trial status	References
1.	Acriflavine	Inhibits premetastatic niche of TNBC by blocking HIF-1α	HIF-1α/LOX	Monotherapy	Preclinical	(97, 320)
2.	As4S4	Inhibits the TNBC metastasis by scavenging ROS and reduce thr transcription level of HIF-1α	ROS/HIF-1α	Monotherapy	Preclinical	(97, 321)
3.	Cardamonin	Inhibits the transcription of HIF-1α	mTOR/P70s6k/HIF-1α	Monotherapy	Preclinical	(97, 322)
4.	Digoxin (DIG)	Blocks the accumulation of HIF-1α and HIF-2α in hypoxic cells and block chemotherapy-induced expression of IL-6, IL-8, and MDR-1, and blocked BCSC enrichment	HIF-1α/VEGF	Monotherapy	Preclinical	(318)
5.	Diallyl Trisulfides (323)	Inhibits the translation level of HIF-1α and inhibits the TNBC metastasis	HIF-1α	Monotherapy	Preclinical	(97, 324)
6.	Elemene (C15H24)	Reduce the stability of HIF-1α	ROS/HIF-1α	Monotherapy	Preclinical	(97, 325)
7.	Ganetespib	Induces the HIF-1α protein degradation and controls the angiogenesis, metabolism, invasion, and metastasis in TNBC	Hsp90/HIF-1α/SDF1/VEGF/GLUT1, HK2/PDK1/ALD1A1, ALD1A3/MMP9 /P4HA1/P4HA2/ANGPTL4/ LICAM/LOX	Monotherapy	Preclinical/II	(97, 326)
8.	Isoliquiritigenin (ILTG)	Inhibits the expression of HIF-1α and VEGF and inhibits the TNBC metastasis	PI3K/Akt/HIF-1α/VEGF//NF-*κ*B	Monotherapy	Preclinical	(327)
9.	Nanoliposomal echinomycin	Blocks the activity of HIF-1α	HIF-1α/VEGF	Monotherapy	Preclinical	(97, 328)
10.	Melittin	Inhibits the transcription of HIF-1α by inhibiting NF-*κ*B expression	HIF-1α/VEGFA/ NF-*κ*B/LDHA	Monotherapy	Preclinical	(328)
11.	Sanguinarine	Induces the proteasomal degradation of HIF-1α	HIF-1α/STAT3 blocker	Monotherapy	Preclinical	(329)
12.	Amphotericin B	Suppress the binding of HIF-1α/p300 complex to HRE	HIF-1α/p300/FIH1 and PI3K/mTOR	Monotherapy	Approved for clinical use	(320, 330)
13.	Apigenin	Inhibits expression of HIF-1α and VEGF	PI3K/AKT/p70S6K1 and HDM2/p53	Monotherapy	II	(331)
14.	YC-1	Inhibits HIF-1α synthesis and blocked angiogenesis and an inhibition of tumor growth	HIF-1α	Monotherapy	Preclinical	(332)
15.	Pleurotin (PX-12)	Inhibits the proto-oncogene (Trx-1) which further blocks the activity of HIF-1α	Trx-1 and thioredoxin 1	Monotherapy	II	(333) (334)
16.	Polyamides	Modulates the HIF-1alpha activity at transcriptional level	HIF-1α	Monotherapy	N/A	(335)
17.	2-phenethyl isothiocyanate (PEITC)	Down-regulates theHIF-1α with reduction of ROS and by induction of Nrf2 signaling	HIF-1α/Nrf2/MMPs 2 & 9/VEGF	Monotherapy	II	(320, 336)
19.	PX-478	Inhibits the expression of HIF-1α and HIF-1 transcription factor activity	HIF-1α/VEGF/GLUT-1	Monotherapy	I	(337)
20.	Silibinin	Inhibits the HIF-1α synthesis and induces the metabolic crisis in triple-negative breast cancer cells by modulating EGFR-MYC-TXNIP axis	mTOR/p70S6K/4E-BP1	Monotherapy	Approved	(338)
21.	Wondonin	Induces the proteasomal degradation of HIF-1α by increasing the interaction of HIF-1α and pVHL	HIF-1alpha/pVHL/ERK1/2//Akt	Monotherapy	Preclinical	(339)
22.	Sulphoraphane	Decrease the HIF-1α and VEGF expression by inhibition of STAT3/HIF-1α/VEGF signaling	HIF-1α	Monotherapy	II	(340)
23.	Cardenolides	Inhibits the expression of HIF-1α and HIF-1 transcription factor activity	HIF-1α	Monotherapy	Approved for clinical use	(341)
24.	DIM (3,3’-Diinolylmethane)	Downregulates the mRNA expression of HIF-1α	HIF-1α/TRAF2/p38 MAPK	Monotherapy	III	(342)
25.	Pseudolaric acid (186)	Inhibits angiogenesis and reduces HIF-1α by promoting proteasome-mediated degradation	JNK/SAPK and p53 and HIF-1α/VEGF/KDR	Monotherapy	Preclinical	(343)
26.	Andrographolide	Suppress COX-2 expression and angiogenesis *via* inactivation of HIF-1α/p300 signaling and VEGF pathway	HIF-1α/p300/VEGF	Monotherapy	III	(344)
27.	Curcumin	Inhibits the expression of HIF-1α and HIF-1 transcription factor activity by degrading ARNT in cancer stem-like cells and reduces the proliferation and metastasis of TNBC cells	Hedgehog/Gli1/HIF-1α	Monotherapy	II	(345)
28.	Echinomycin	Inhibits HIF-1α transcriptional activity of primary and metastatic TNBC cells	HIF-1α	Monotherapy	Rejected after Phase II trial	(328)
29.	Flavopiridol (alvocidib)	Inhibiting HIF-1α gene transcription	HIF-1α	Monotherapy	III	(346)
30.	GA and analogs	Induces the proteasomal degradation of HIF-1α	HIF-1α/Hsp90	Monotherapy	II	(347)

### 5 Conclusion and future prospective

A preponderance of evidence supports the notion that different histological and molecular subtypes of TNBC signify its heterogeneity and aggressiveness. Several genetic and transcriptomic alterations define each subtype of TNBC, and they can be potentially targeted for a unique therapy. Recent advancements in targeting hypoxic-tumor microenvironments by suppressing HIF-1α transcription and oxidative phosphorylation have yielded promising results. Besides, anti-angiogenesis inhibitors and hypoxia-activated pro-drugs gained a lot of attention. Moreover, recent studies confirmed that TNBC also causes hypoxia-dependent genetic changes in DDR pathways, which suggests the possibility of predictive biomarkers. Combining DDR inhibitors with other therapy, including radiotherapy, chemotherapy, immunotherapy, PDT, and adjuvant therapies, can optimize their efficacy in TNBC treatment. TNBC also characterize by complex immunological landscape vulnerability through defects in the DDR pathway, which induces high TMB, anti-tumor immune suppressive features, as well as adaptive immune resistance *via* the expression of corresponding inhibitory ligands against immune checkpoints such as the PD1-PDL1 interaction. Therefore, DDR deficiencies offer potential therapeutic leverage for TNBC treatment by combining DNA/DDR-targeted therapies with cytotoxic anti-tumor immune cells, leading to favorable immune effects. Combining immune-checkpoint inhibitors, chemotherapy, and radiotherapy with HIF-1α inhibitors or its downstream target inhibitors like Trk, PI3K, PARP, CAIX etc., maybe a significant potential to match the high standard of clinical benefit in TNBC. In addition, combining these inhibitors with emerging antibody-drug conjugates, cancer vaccines, or adoptive cell therapy followed with existing treatments may be a significant step towards precision therapy and extend overall clinical benefits. Therefore, to determine the solid TNBC combination therapy regimens, it is pertinent to access the immune-molecular expression of HIF-1α and its associated mutational analysis in hypoxic TNBC. Although, HIFs have already been largely explored, but their downstream effector signaling, as well as other pathways like MYC, TP53, and KRAS, should be further explored in the surge of potential therapeutic targets. A more in-depth understanding of the TNBC hypoxic microenvironment, its molecular nature and its effect on tumor prognosis and survival will surely help in early detection and accurate treatment. 10,000 human genome projects will definitely aid in designing precise medicine based on the individual genome as well as tumor specificity.

## Author contributions

Conceptualization: NS and SU; writing and original draft preparation: NS, SU, RSS, and PKP; writing—review and editing: NS, SU, RSS, and RS; visualization: NS, SU, RSS, and RS; supervision and critically reviewed: NS, SU, RSS, and RS. All authors contributed to the article and approved the submitted version.
